# Random-forest model for drug–target interaction prediction via Kullbeck–Leibler divergence

**DOI:** 10.1186/s13321-022-00644-1

**Published:** 2022-10-03

**Authors:** Sangjin Ahn, Si Eun Lee, Mi-hyun Kim

**Affiliations:** 1 Gachon Institute of Pharmaceutical Science and Department of Pharmacy, College of Pharmacy, Gachon University, 191 Hambakmoeiro, Yeonsu-gu, Incheon, Republic of Korea; 2 Department of Artificial Intelligence, Ajou University, Suwon, 16499 Republic of Korea

**Keywords:** Chemocentric, 3D Molecular Fingerprint, 3D Similarity, Drug–Target Interaction Feature, Nonparametric Density Estimation, Kullbeck–Leibler Divergence, Machine Learning

## Abstract

Virtual screening has significantly improved the success rate of early stage drug discovery. Recent virtual screening methods have improved owing to advances in machine learning and chemical information. Among these advances, the creative extraction of drug features is important for predicting drug–target interaction (DTI), which is a large-scale virtual screening of known drugs. Herein, we report Kullbeck–Leibler divergence (KLD) as a DTI feature and the feature-driven classification model applicable to DTI prediction. For the purpose, E3FP three-dimensional (3D) molecular fingerprints of drugs as a molecular representation allow the computation of 3D similarities between ligands within each target (Q–Q matrix) to identify the uniqueness of pharmacological targets and those between a query and a ligand (Q–L vector) in DTIs. The 3D similarity matrices are transformed into probability density functions via kernel density estimation as a nonparametric estimation. Each density model can exploit the characteristics of each pharmacological target and measure the quasi-distance between the ligands. Furthermore, we developed a random forest model from the KLD feature vectors to successfully predict DTIs for representative 17 targets (mean accuracy: 0.882, out-of-bag score estimate: 0.876, ROC AUC: 0.990). The method is applicable for 2D chemical similarity.

**Supplementary Information:**

The online version contains supplementary material available at 10.1186/s13321-022-00644-1.

## Introduction

Several machine learning (ML)-based methods have been widely applied in chemo-informatics-related areas. From the classical Bayesian approach to recent deep-learning technologies, novel molecular representations and descriptors for characterizing molecules are vital to computer-aided drug discovery [1–4]. In drug–target interaction (DTI) prediction, versatile featurization methods and learning methods have been developed from a “network analysis based on experimental DTI information as feature vectors” to a “deep learning-based DTI prediction from diverse representation (protein or gene sequences, drug structures, explicit target-drug binding structures)” [5–12]. While former studies mainly used independent feature vectors for each representation, recently reported studies used the featurization of “target–drug binding complexes” (e.g., IFP, PLIP, SIFt, and SMPLIP) [13–16]. Despite the efficiency of the above mentioned featurization, explicit binding poses are not applicable to an undefined and endless number of target proteins, in particular, epigenetically modulated proteins (different from native proteins of ca. 20,000 human genomes), their mutants, and fusion proteins. Thus, we judge “chemical features” remain effective for DTI prediction beyond the explicit binding. Chemocentric methods using chemical features can describe both indirect DTIs (via controlling a target without physical binding with a drug) and direct DTIs (via a binding complex of drug-target). Historically, both chemocentric DTI prediction and quantitative structure–activity relationship (QSAR) studies have been conducted using molecular descriptors and similarity scores [4, 17–22]. Reported chemical feature-based DTI studies have commonly focused on known drugs and their properties, such as toxicity, repositioning, or polypharmacology [5, 8, 23–25]. In those studies, two-dimensional (2D) similarity methods (with 2D features) are typically used [5, 6, 8, 24] because they are more economical than three-dimensional (3D) similarity methods (with 3D features) [17, 21]. While both 2D methods and synthetic chemists’ intuition commonly use 2D structures of chemicals and drugs, 3D methods can provide another view (consequently, for new knowledge), which is not accessible by synthetic chemists’ intuition and is distinct from 2D DTI prediction [26]. Meanwhile, despite the availability of 3D descriptors such as E3FP [18], 3D chemical similarity has rarely been applied to DTI prediction [25]. Moreover, reported DTI prediction studies using similarity transform similarity scores into statistical values in a probability density distribution (e.g., p- and E-values, Z-score) and compare the values with a cutoff [5, 23–25]. The schemes for DTI prediction developed so far do not focus on modeling the heterogeneity of probability densities [5–16, 23–25]. In this study, we used a heterogeneous probability density distribution of 3D similarity vectors to obtain a reliable DTI predictive model. In particular, we incorporated a nonparametric density model into our previous Kullbeck–Leibler divergence (KLD)-based quantifying method [26], which observes ligands from the viewpoint of candidate targets, such that multiple KLD measurements can be performed to describe a drug (query).

**Fig. 1:**
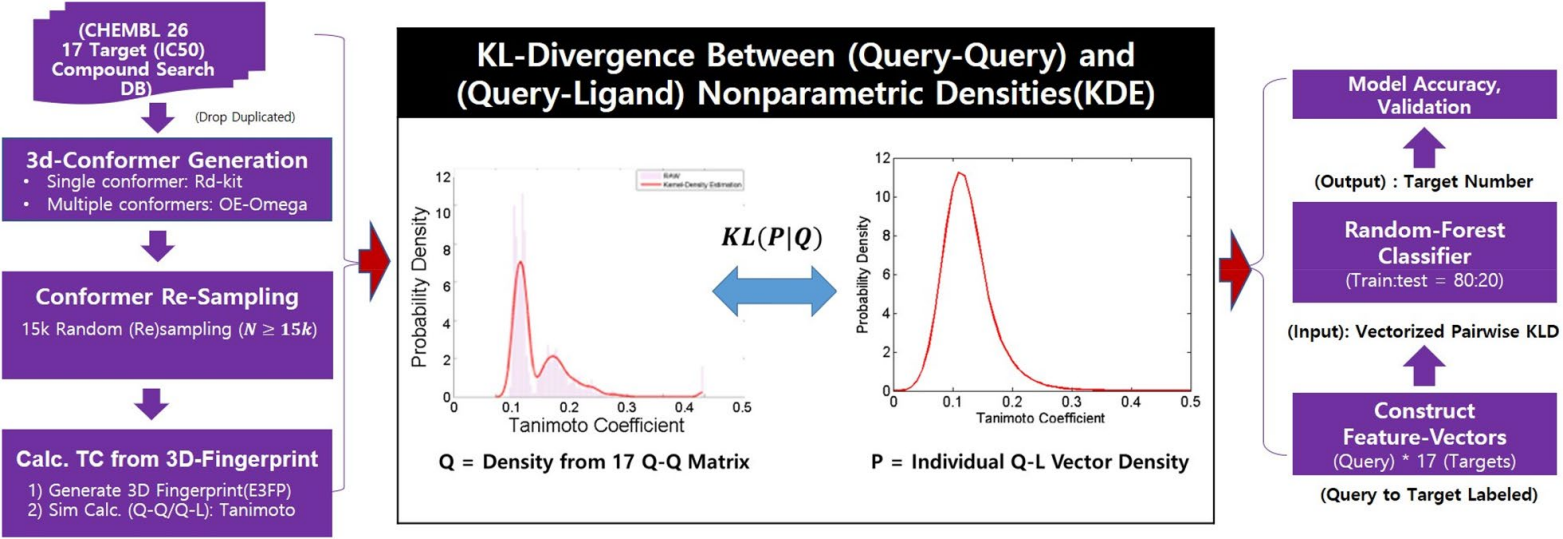
Overview of this study. Kullbeck–Leibler divergence (KLD) between chemical similarity distributions (of the Q–Q matrix and the Q–L vector) provided feature vectors for drug target interaction (DTI) prediction. The distributions were generated through kernel density estimation (KDE) as a nonparametric density model, which is quite distinct from the Gaussian distribution defined by the mean and standard deviation of a sample

Feature engineering is essential for ML-based drug discovery. Recently, ML-based DTI detection (descriptive and predictive) and ML-aided drug discovery studies have contributed positively to the feature engineering of molecular data. The performance of ML approaches relies on their molecular representations. These ML approaches require the perfect transferability of molecular information during molecular representation, similarity scoring, and learning. Hence, we attempt to link our 3D similarity-based quantitative method [26] with an ML algorithm to predict whether each query belongs to a candidate target. Furthermore, we introduce chemocentric assumptions and the 3D similarity used in our previous study [26] First, based on E3FP (3D radial molecular fingerprints), pairwise similarities are calculated between ligands within each target (Q–Q matrix) and between a query and a ligand (Q–L vector) for DTIs. Second, 3D similarity vectors (Q–L) and matrices (Q–Q) are probabilistically modeled to describe the uniqueness of targets (Q–Q) and to quantify ligand-specific information for DTIs (Q–L). Finally, the KLD works as a “quasi-distance” among the density models, and KLD as a novel DTI feature vector is successfully extended to the DTI prediction model (Fig. 1).

## Methods and materials

### Dataset and data preparation

We obtained biological activity data from the publicly available CHEMBL 26 database [27]. The database contains information regarding more than 200 single-protein targets and their chemical and genomic properties. In this study, we used 17 targets selected from a benchmark paper [28]. The downloaded information table contains a list of smiles from CHEMBL26 databases, which describes the “molecule name,” “SMILES,” and “IC50” value for each listed CHEMBL ID. Duplicate items were removed to avoid sampling bias. We focused on 26,452 ligands and 2.976 million conformers. Data handling and algorithm computing were conducted using Python and its modules. The 3D conformers were generated under conditions reported by OpenEye Omega [29, 30] and under RDKit.

### Three-dimensional fingerprinting and ligand pairwise 3D molecular similarity.

All the original ligand spaces from the 17 targets were randomly resampled using 15,000 conformers. The size of each molecular conformation was limited to 15,000 conformers. Thus, we attenuated the problems of dimensionality and data imbalance. Ten-time tests were performed to determine the stability of random sampling. We confirmed that changes in “random seeds” realistically provided stability to the similarity score density structure. Among the numerous descriptors for molecular representation, E3FP was selected to effectively describe the 3D structure of the molecules. Each 3D fingerprint depicting a ligand conformer was encoded using the E3FP in the RDKit library. In other words, E3FP generated 3D molecular fingerprints in the RDKit library, and each 3D conformer was converted to the RDKit format to calculate the similarity scores among the ligands. The 3D coordinates of each conformer expressed in sdf format were converted and encoded to a sequence of bit-vectors composed of 1024 “0” and “1”. Subsequently, the similarity scores were calculated by comparing the bit-vectors. This bit = vector-based similarity score calculation is computationally less expensive than the maximum common substructure-based approach or shape-based approach (Openeye Shape Toolkit) and retains the 3D conformation [30, 31].

### Q–Q matrix

The Q–Q matrix contains the pairwise similarity scores of all the ligands belonging to a candidate target. Its dimensions were up to 15,000 × 15,000. Let ${\mathrm{M}}_{Q-Q}$ be the similarity matrix obtained from 17 independent targets; its elements ${\mathrm{a}}_{1, 1}, \dots , {a}_{15,000, 15,000}$ are set of pairwise similarity scores of ligands belonging to a certain target. These matrices can be regarded as benchmarks for measuring target-specific (collective and global) information. The descriptive statistics (density information) of the Q–Q elements are expected to differ among the targets. However, it preserves the stability of ligand sampling.

$$M_{{Q{-}Q}} = \left[ {\begin{array}{*{20}l} {a_{1,1} } & {a_{1,2} } & \cdots & {a_{15,000} } \\ \cdots & {} & {} & {} \\ {a_{15,000,1} } & {} & {} & {} \\ \end{array} } \right]$$

### Q–L vector

Next, we prepared the Q–L similarity vector to express and measure the interaction between a (certain) query and the candidate target. These vectors preserve ligand-specific information, whose descriptive statistics differ based on the ligand, and the size of each vector is 1 × 15,000 (maximum). While a Q–Q matrix indicates the comparison of ligands “within” a target, a Q–L vector can be obtained from each column vector of the pairwise similarity matrix “between” two targets. This can be referred to as a query’s “observation” in terms of the candidate target’s view. Each vector is comparable to each matrix when they share a common ligand.

$$M_{Q - L} = \left[ {\begin{array}{*{20}l} {b_{1} } \\ {b_{2} } \\ \cdots \\ {b_{15000,1} } \\ \end{array} } \right]$$

### Probability density function of each vector space

**Fig. 2:**
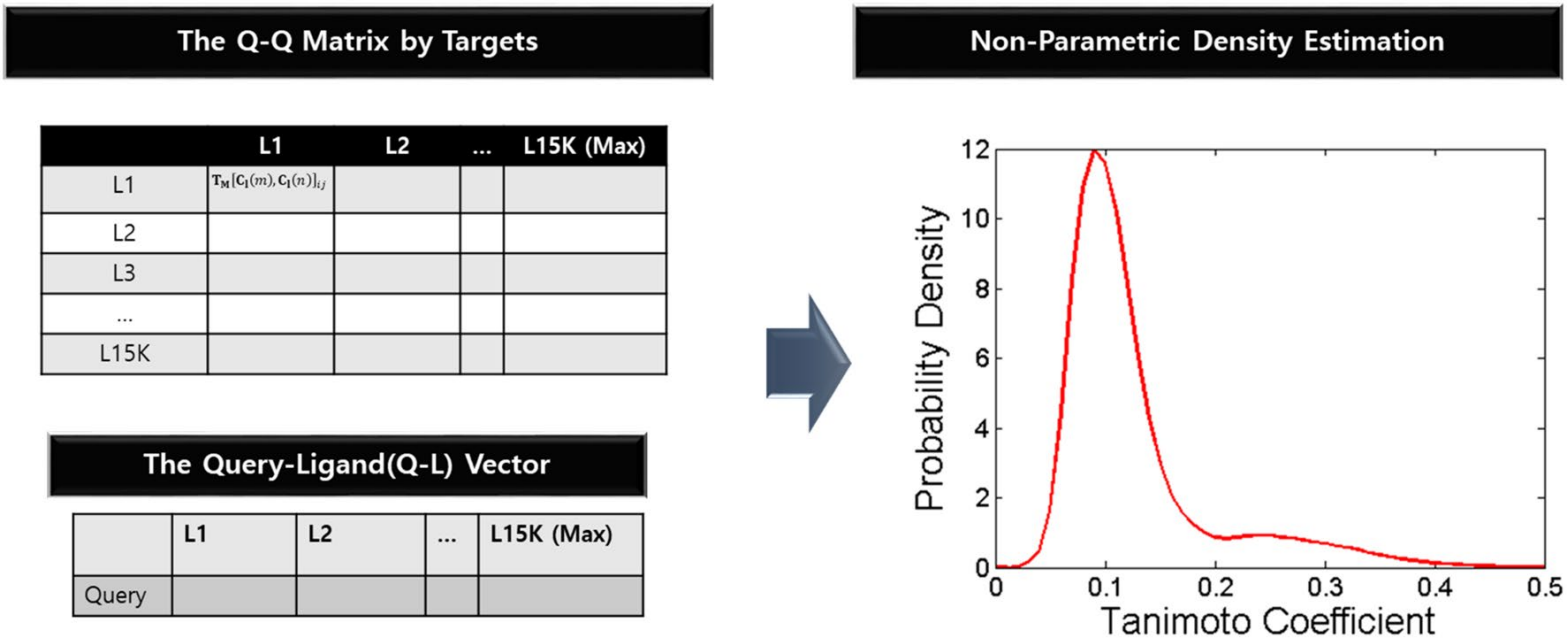
Transformation of Q–Q matrix or Q–L vector into KDE distribution

In our experiment, we considered probabilistic information reflecting the target representation and ligand-to-target interaction. Generally, the shapes of the Q–Q and Q–L matrices, whose number of ligands depends on the target, are different. A method to unify and structure their information is to use their probability density functions. We determined the distributions of both the matrix and vectors (Fig. 2). Each matrix density function (pdf) projects unique representations of each target. Specifically, the tail shape, symmetry, bias, and sharpness differ between the targets. Similarly, the vector density reflects the information obtained from the query (ligand)–target interaction. Each probability density function is represented by the function y = p(x), and q(x) for each x-axis point divides the interval [0, 1] into 100 equal sections. After being combined with the information metric, these probability distributions p(x) and q(x) are the main components that constitute the feature vector of our classification model.

### Kernel density estimation

A well-known nonparametric density estimation method, kernel density estimation (KDE), was selected to estimate the probability density function [32–35]. The built-in likelihood function was maximized to estimate the probability distribution function for the data obtained. For probability distributions obtained via KDE, the probability of points on each x-axis can be obtained as follows:

$$\widehat{p}\left(x\right)\propto \sum_{i=1}^{100}kernel\left(\frac{x-{x}_{i}}{\left(Bandwidth\right)}\right)$$

In KDE, when the input matrix and vector are constructed, the estimation is performed using a Python script, and a 1 × 100 vector containing each pdf value is output. Scipy’s Python package [36], which automatically selects the optimal bandwidth for KDE, allows us to apply a Gaussian kernel. We confirmed that the density structure in this study rarely depends on the KDE methodology and bandwidth. Both Silverman’s and Scott’s methods yielded satisfactory results. Moreover, such a nonparametric approach provides flexible and stable results regardless of the experimental environment, and also provides results with fewer density estimation errors. In this study, KLD was used to calculate the “difference” between the estimated density functions from each Q–Q matrix and Q–L vector. The difference between the density models is interpreted as a measure of a query’s interaction with a target.

### KLD

KLD is a relative entropy that measures whether two probability density functions are different or equal [37–39]. Lower KLD values imply a higher similarity between two density functions and vice versa. The KLD values of each query serve as a metric to measure the relative similarity of the query against possible targets. The feasibility of a query belonging to a target was determined by comparing the KLD values of the mapped Gaussian mixture model [26]. In other words, KLD can be computed from the pdf in [0, 1]. Let q(x) be the 17 Q–Q densities postulated to be fixed to describe the representative characteristics of certain targets. Our observation of a ligand toward a candidate target, p(x), was obtained from the Q–L vector. We used KLD to measure the degree to which the Q–L vector density (p(x)) differs from the 17 matrices (i.e., the candidate target density, q(x)). The divergence between the query and query similarity density function and the Q–L density measures the magnitude of the difference between a query and a candidate target and illustrates the process by which KLD is calculated. To calculate the KLD directly, a small number is added to the functional value of both p(x) and q(x) by considering the point where q(x) is zero. Let P = {p_1,…, p_17}, q = {q1,…, Q_17}. Subsequently, the KLD is calculated as follows:

$$\mathrm{KL}\left(\mathrm{p}|\mathrm{q}\right)= {\sum }_{x}p\left(x\right) ln\frac{p(x)}{q(x)}*dx+ {\sum }_{x}\left(q\left(x\right)-p\left(x\right)\right)*dx$$

Seventeen KLD values and the ligands (query) from the candidate targets were obtained. The divergence of q from p approximates to a minimum if the Q–L density is similar to the Q–Q density, such that the value provides a measurement of the distance between a query and several candidate targets for a certain query. In general, a small KLD value suggests that a query has a high similarity to the target, which corresponds to whether a certain query belongs to the target and vice versa. In random-forest (RF) models, individual KLD values become a feature of each query that describes the measurement from the viewpoint of the candidate target. Finally, we obtained a labeled vector with a divergence measuring 1 × 17 from each ligand (query) for the RF classifier.

### RF classifier

The RF models, which comprise an ensemble of several decision-tree models, were selected for this nonparametric methodology [40, 41]. The well-known classification and regression tree algorithm can be easily extended to large-scale data [42–46]. We considered a feature measuring 17 × 1 for each ligand. Each query was labeled with its target number (1, 2, …, 17), and each decision process of the RF algorithm was generated by comparing the KLD values of each query. For the 17 × 1 feature vector, which comprises KLD values, the target was predicted by combining the decisions from individual features. In other words, by measuring the KLD values, the RF classifier was instructed to determine whether a query is suitable for a target. The RF classifier implicitly facilitated correspondence in the value of KLD between such a similarity density and an indirect difference between a query and a candidate target. The optimal parameters for our RF model were automatically adjusted using the scikit-learn package [36]. In our experiment, the RF predicted the most probable target from the KL-divergence measured by each candidate target. Combining nonparametric density estimation and KLD, the RF model can provide a solution to the DTI prediction problem.

## Results and discussion

In this study, the probabilistic modeling of chemical similarity was performed to describe the features of a certain ligand (drug) in the RF model. First, similarity information was implemented into the KLD equation via nonparametric density estimation. Second, the calculated KLD values enabled quantitative comparisons between targets and a ligand (query). Finally, the RF classifier was built using the KLD feature vectors for DTI prediction. In this section, we present the results of our study, including the predictive power of the RF classifier and the results of feature analysis.

### Representation of targets and ligands via nonparametric probability distribution model

**Fig. 3:**
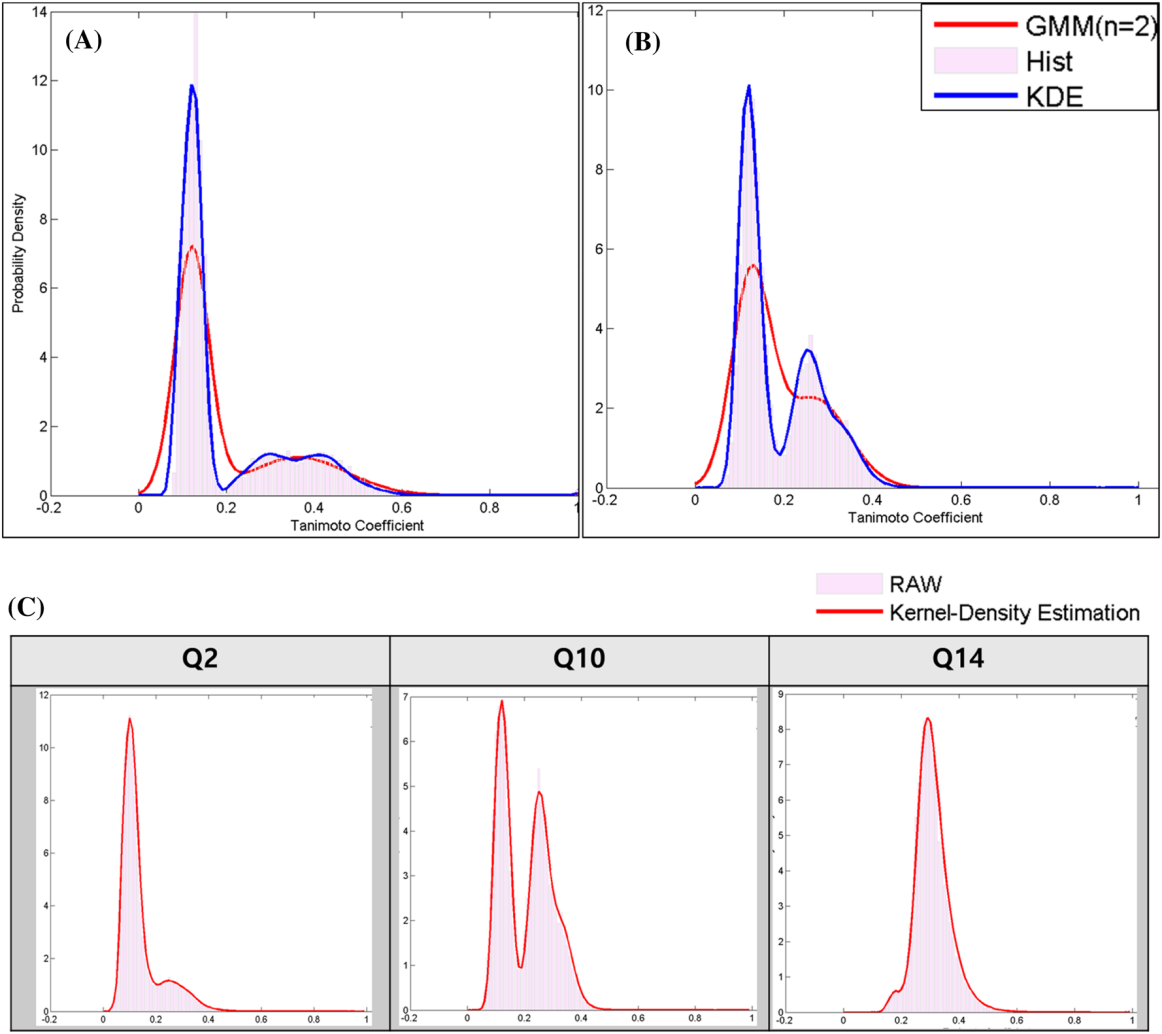
Comparison of their probability densities with a 3D similarity distribution (of the Q–Q matrix). **A** 3D similarity histogram and probability densities, GMM (n = 2) and KDE of sigma opioid receptor (Q3), **B** 3D similarity histogram and probability densities, GMM (n = 2) and KDE of fibroblast growth factor receptor 1 (Q10), and **C** Heterogenous 3D similarity distribution between three targets, heat shock protein 90 (Q2), fibroblast growth factor receptor 1 (Q10), serine threonine-protein kinase mTOR (Q14). X-axis: 3D similarity (Jaccard–Tanimoto coefficient), Y-axis: relative frequency

Herein, we introduce the terminology “target class.” Because a Q–Q matrix is obtained from a group (a class) of ligands sharing a target protein, the matrix characterizes a target using its ligand information to represent the target under the chemocentric assumption [26, 47]. Thus, to conveniently name the group of a specific Q–Q matrix, we named each group of the target class with its target name. The similarity information of the target classes was represented by a nonparametric probability distribution model of the respective Q–Q matrix. Whereas many classes were slightly skewed but similar to a Gaussian distribution, some classes differed significantly from the Gaussian distribution, e.g., the sigma opioid receptor (Q3) of Fig. 3A, fibroblast growth factor receptor 1(FGFR1) of Fig. 3B shows that the probability density of each target class can be severely asymmetric and skewed, rendering it difficult to assume structural consistency. Notably, FGFR1 (Q10), which contains > 1000 ligands, cannot be fitted well to a Gaussian model. Without structural (e.g., Gaussian and gamma) assumptions on similarity data, nonparametric density estimation provides more flexibility and less information loss than previous Gaussian mixture models (GMM) [26]. As shown in Fig. 3 and Additional file 1: Figure S1, the KDE perfectly fits the unique distribution of the respective target classes. The results in Fig. 3 are different from most of the studies involving chemical similarity, which assume that the similarity distribution is a Gaussian distribution [48]. Because the composition of the target classes differs based on the orthosteric ligands, allosteric ligands, and non-direct binding regulators, their distributions are dissimilar to each other and do not conform to the Gaussian distribution. Thus, we conclude that the KDE distribution is a more reasonable method than the parametric GMM for describing chemocentric DTI prediction.

In addition to the representation of targets, the relationship between a specific ligand (drug) and a target was represented in the KDE model of the respective Q–L vector. The data dimensions between the Q–L vectors differed significantly due to the different number of ligands within a target class (the maximum size of a Q–L vector was 15,000). However, the KDE provided a stable (sufficiently good) density distribution regardless of the dataset size. The probability density distribution describes the respective pair’s characteristics (a ligand and a target class) and allows a comparison between the “target class–drug” pairs. In other words, the KDE distributions of the Q–L vectors imply DTIs. Whereas a pairwise comparison within a fixed target class is facile and reasonable (e.g., Drug 1⎼Target 1 (D1–T1) vs. D2–T1), the target-wise comparison (D1–T1 vs. D1–T2) or a cross-comparison (e.g., D1–T2 vs. D2–T1) is difficult. Notably, the difference between the characterized targets (confounding effects) should be adjusted for the pair comparison. Thus, pair comparisons should be generalized and quantified across the targets for DTI prediction. To perform this, we used the KLD as an information measure or relative entropy. Because the KLD measures the difference between two statistical or probabilistic distributions [26], it can provide the similarity information of any “target class–drug” pair considering the characteristics of the target in the pair. This allows us to incorporate the characteristics of targets $q\left(x\right)$ into the pairwise comparison, $p(x)$ (the equation in Subsection 2.7 of the Materials and Methods section) for cross-comparison (e.g., D1–T2 vs. D2–T1) or target-wise comparison (D1–T1 vs. D1–T2). In other words, the probability density function $q\left(x\right)$ is the KDE model of the respective Q–Q matrix. Therefore, the cross- or target-wise comparison changes the $q\left(x\right)$ across targets. Meanwhile, the three comparisons require only two $q\left(x\right)$ generated from two Q–Q matrices and three $p(x)$ generated from three Q–L vectors. Thus, paradoxically, the “extraordinary” density distribution (showing severe asymmetry, skewness, and a fat tail) is preferred to verify the practicability of this method, where the information entropy (KLD) is calculated and used without considering a statistical rule or a cut-off (e.g., comparison between the significance and *p*-value under the null hypothesis).

$$\mathrm{KL}\left(\mathrm{p}|\mathrm{q}\right)= {\sum }_{x}p\left(x\right) ln\frac{p(x)}{q(x)}*dx+ {\sum }_{x}\left(q\left(x\right)-p\left(x\right)\right)*dx$$

### KLD as DTI descriptor

To our knowledge, chemical similarity is not popularly used as a single feature in DTI prediction [5–12, 24, 25]. Thus, we investigated a chemo-centric DTI descriptor able to give better discriminative power than the similarity scores of one drug for multiple targets. As mentioned above, the probability densities of target classes vary considerably (Fig. 3). Thus, when a new drug is compared with multiple target classes, the relative location of a similarity score in the probability densities, like the E-value of SEA, is more important than the highest score (e.g., max of Tc) extracted from similarity scores [47]. Meanwhile, the KLD calculation includes the relationship between all ligands of a target class based on the q(x) of the Q–Q matrix (target-specific information) and the relationship of a query drug with a target class based on the p(x) of the Q–L vector (ligand-specific information). The KLD value doesn’t reply on either the highest similarity score or the cut-off (of similarity score, statistical Z-score, p-value, E-value), but describes the relative similarity between a new drug and a target class. When a new drug shows a smaller KLD value for a specific target class than those for other classes, we predict the DTI of the drug–target pair. This point renders KLD values as a new chemo-centric DTI descriptor distinct from any molecular descriptor or similarity score (one KLD value; relationship of one drug–target pair vs. one similarity value; that of one drug–drug pair vs. one molecular descriptor; information regarding one drug). Thus, we attempted to determine the potential of distribution divergence as a DTI descriptor. As mentioned in the “KLD as DTI descriptor” section, the KDE distribution showed a suitable proxy representing the q(x) of the Q–Q matrix and the p(x) of the Q–L vector. The divergence quantifies the DTI prediction between an individual drug and target class by comparing q(x) and p(x).

The probability density q(x), which identifies the relevance between ligands “within” a target class, provides target-specific information. Thus, notably, both individual (ligand–target) density and collective (target–target) density can be compared via the KLD. For collective (target–target) density, we could examine the target–target density with pairwise target analysis (Table 1). In other words, the KLD values between paired target classes (Q–Q vs. Q–Q matrix) were calculated. In addition, the reverse divergence quantity was calculated by substituting q(x) and p(x) in the reverse position (Table 1). The dual quantities (KLD and reverse KLD) describe the relevance between the target classes. The pair with lower divergence suggests that the target classes exhibit similar distributions, implying similar characteristics between them. The KLD measures the extent to which a query (drug or target) is different from a target. Thus, we spontaneously applied this notion to the DTI classification model.

**Table 1:** KLD between target pairs (Q–Q matrix vs. another Q–Q matrix, 17 × 17 target)^a^

T	1	2	3	4	5	6	7	8	9	10	11	12	13	14	15	16	17
1	0.00	0.11	0.57	0.12	0.02	0.03	0.38	0.13	0.15	0.47	0.29	0.11	**2.54**	0.09	0.04	0.02	0.03
2	0.11	0.00	0.50	0.22	0.16	0.21	0.13	0.41	0.08	0.42	0.18	0.33	1.85	0.32	0.13	0.16	0.17
3	0.36	0.55	0.00	0.20	0.43	0.41	1.13	0.33	0.89	0.27	1.17	0.37	1.15	0.29	0.40	0.27	0.48
4	0.09	0.24	0.23	0.00	0.13	0.14	0.66	0.10	0.43	0.17	0.66	0.06	1.38	0.06	0.19	0.07	0.14
5	0.02	0.18	0.63	0.17	0.00	0.01	0.40	0.10	0.15	0.66	0.27	0.11	**2.90**	0.08	0.03	0.04	0.01
6	0.04	0.23	0.69	0.21	0.01	0.00	0.46	0.10	0.18	0.77	0.28	0.13	**3.19**	0.09	0.04	0.05	0.02
7	0.35	0.11	0.66	0.53	0.40	0.45	0.00	0.80	0.09	0.79	0.09	0.71	**2.07**	0.68	0.33	0.44	0.41
8	0.18	0.55	0.54	0.14	0.17	0.15	1.18	0.00	0.74	0.49	1.03	0.03	**2.49**	0.01	0.24	0.09	0.17
9	0.13	0.09	0.78	0.38	0.13	0.16	0.10	0.42	0.00	0.86	0.03	0.39	**2.98**	0.36	0.11	0.21	0.14
10	0.40	0.61	0.31	0.16	0.48	0.48	1.28	0.32	1.00	0.00	1.33	0.27	0.87	0.27	0.52	0.28	0.49
11	0.28	0.26	1.08	0.68	0.25	0.25	0.16	0.61	0.04	1.45	0.00	0.64	**4.21**	0.57	0.18	0.38	0.27
12	0.12	0.39	0.47	0.07	0.12	0.12	0.86	0.03	0.53	0.34	0.76	0.00	**2.11**	0.02	0.22	0.07	0.11
13	**3.43**	**3.99**	**2.22**	**2.39**	**3.49**	**3.47**	**4.80**	**2.71**	**4.52**	**1.74**	**4.98**	**2.51**	0.00	**2.72**	**4.08**	**3.30**	**3.45**
14	0.12	0.40	0.45	0.07	0.12	0.11	0.95	0.01	0.58	0.38	0.84	0.02	**2.15**	0.00	0.18	0.05	0.12
15	0.05	0.17	0.72	0.28	0.04	0.04	0.41	0.19	0.15	0.84	0.26	0.23	**3.31**	0.17	0.00	0.07	0.06
16	0.03	0.18	0.45	0.08	0.04	0.05	0.56	0.08	0.30	0.38	0.49	0.07	**2.30**	0.05	0.06	0.00	0.05
17	0.03	0.19	0.75	0.21	0.01	0.02	0.43	0.11	0.15	0.73	0.28	0.10	**3.21**	0.10	0.05	0.05	0.00

^a^The lower KLD values indicate that the pairs has similar distribution

**Fig. 4:**
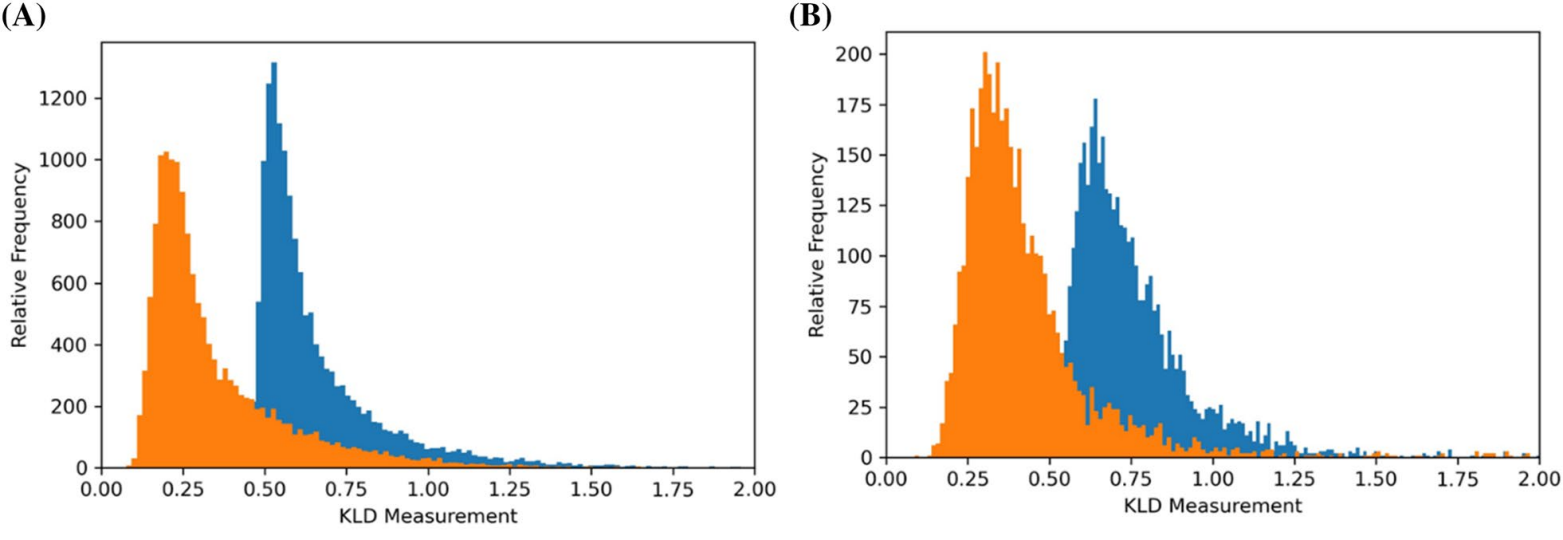
Comparison between 2 and 3D based KLD values between target (Q-Q) density and ligand distribution (Q-L). X-axis: KLD value, Y-axis: relative frequency. The Q-Q density in the orange colored histogram is FGFR1 (Q10), that of the blue colored is mTOR (Q14) **A** KLD measurement from 3D-similarity (E3FP fingerprints of conformers from FGFR1 and mTOR ligands), **B** KLD measurement from 2D-similarity (Morgan fingerprints of ligands from FGFR1 and mTOR)

Furthermore, the results in Fig. 4 show that the KLD values are applicable to both 2D and 3D similarity-based DTI predictions. Because the current 2D methods can be used in the DTI network and QSAR of multiple classes without causing the uncountable data point issue (conformational sampling), the utility of the KLD as a DTI descriptor may not be as significant in 2D methods as it is in 3D methods. By contrast, if a novel target contains only a few ligands, then 3D similarity methods can provide more enriched information regarding the target using conformational ensembles, and our method can assist known 2D methods and other DTI prediction. Moreover, as shown in our previous study [26], although 2D methods are more cost-effective in terms of on-target (primary target) predictions than 3D methods [17, 21], the 3D similarity remains crucial for the in silico target screening of unprecedented drugs [49] because (1) novel, unprecedented drugs exhibit extremely low 2D similarity to known drugs [50–52], (2) novel pharmacological profiles of drugs are more frequently determined using similar 3D off-target predictions [53], and (3) realistic drug properties can be generated from their factual and flexible 3D structures (conformers) [23, 54, 55].

### DTI prediction of RF classifier

**Fig. 5:**
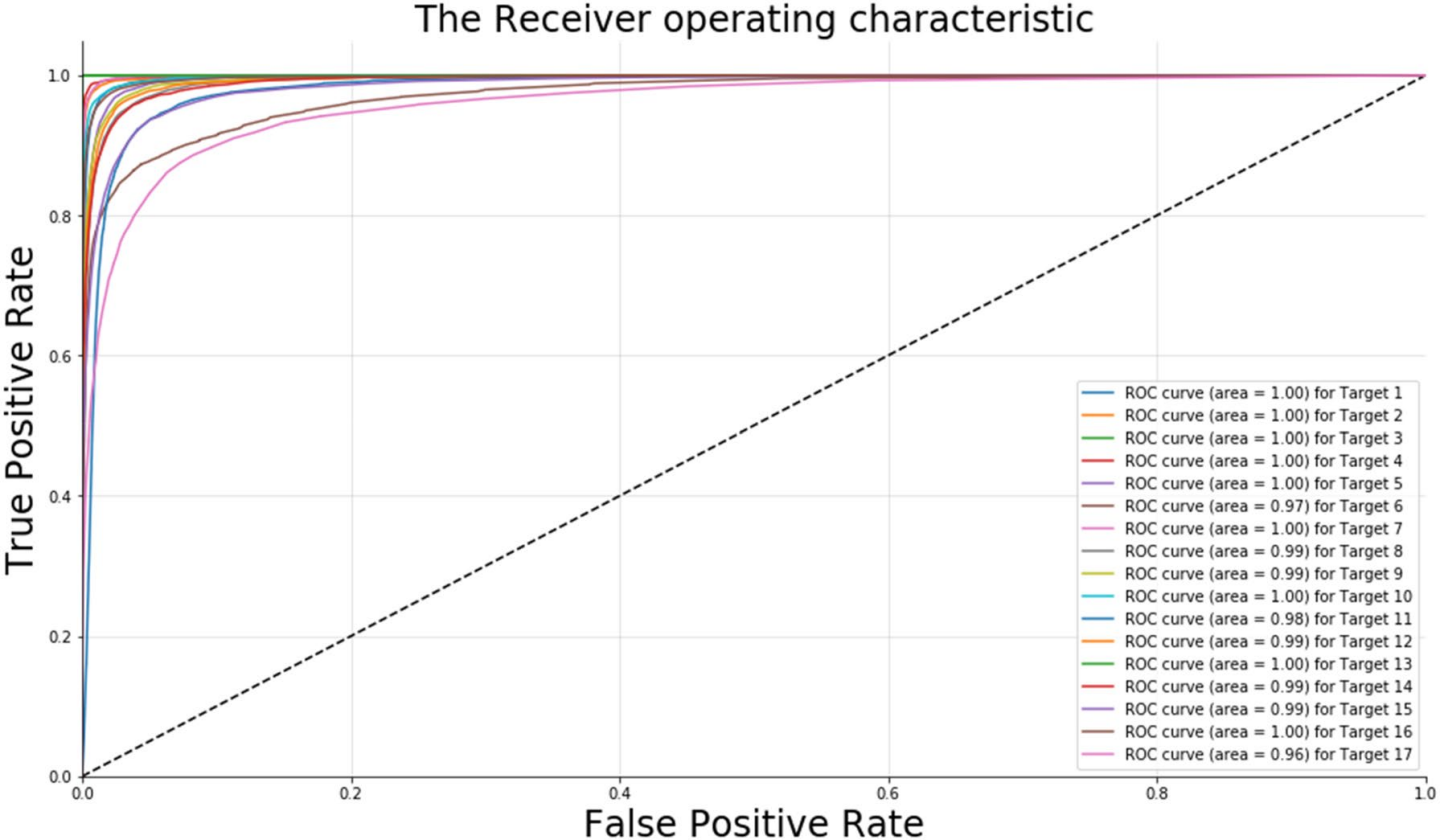
The ROC curves of the test data show DTI prediction performance. X-axis: false positive rate; Y-axis: true positive rate. Each line indicates the respective target class with AUC values

**Fig. 6:**
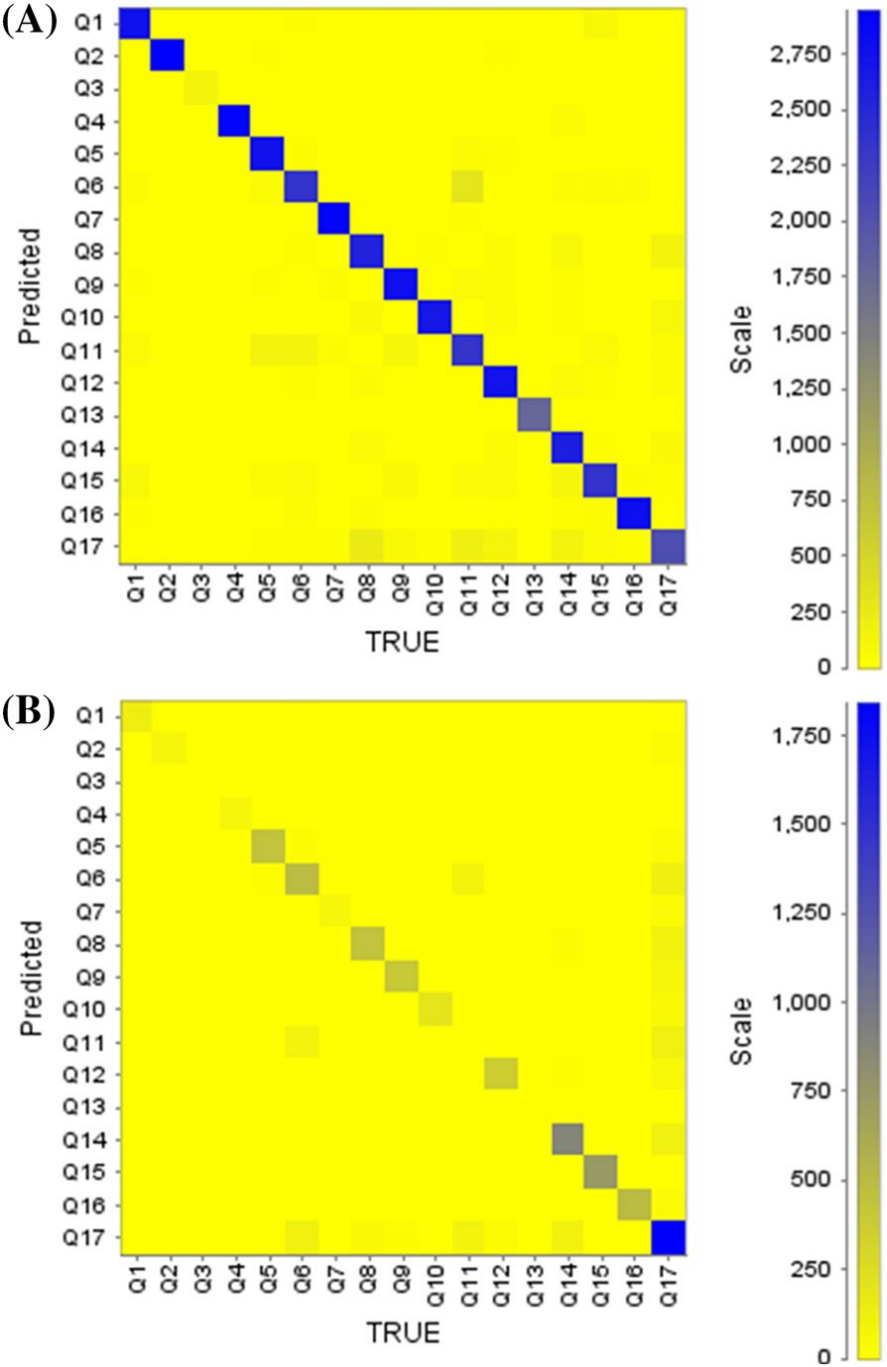
The confusion matrix of the test set showing DTI prediction performance. X-axis: True DTI; Y-axis: Predicted DTI. **A** 3D KLD-RF classifier, **B** 2D KLD-RF classifier

**Fig. 7:**
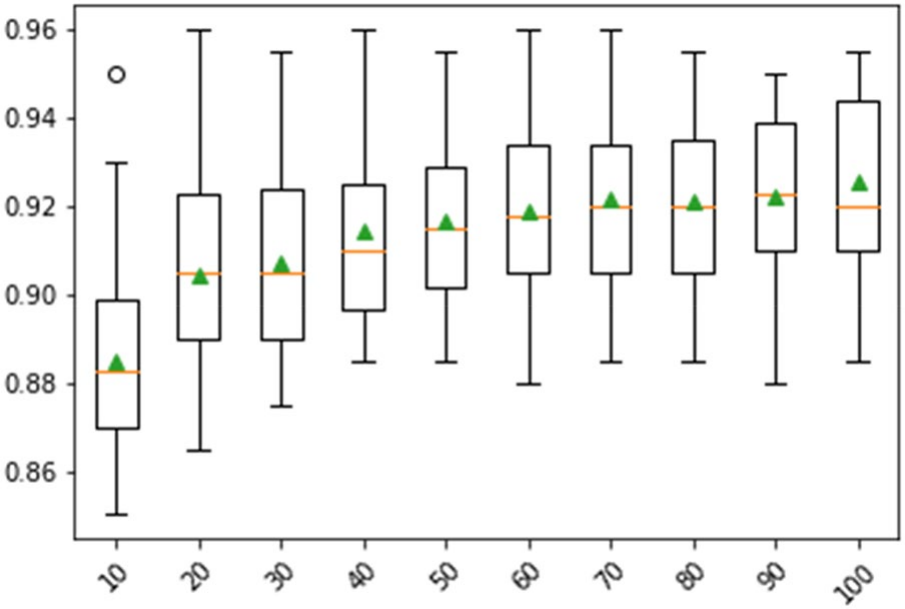
The box plots showing DTI prediction performance. X-axis: percentile rank of KLD features; Y-axis: average precision

A binary classification model was constructed using the KLD for the DTI prediction of individual query drugs. Predictive models from divergence-coordinated features were investigated based on training (75%) and test (25%) datasets. The RF algorithm showed reliable statistical performance and is a desirable classifier for DTI prediction (Table 2, Figs. 5, 6, 7). Despite the imbalanced number of ligands between different targets, the ensemble learning indicated acceptable precision and recall in the test set for every target (Table 2). Epidermal growth factor receptor (Q17), which shares some ligands with all targets except for Q4 and Q13, showed lower performance than that of other targets. Similarly, Q11 also shared some ligands with twelve targets. Based on fivefold cross-validation, the average validation accuracy was 0.88. Moreover, we visualized our model by constructing both the receiver operating characteristic (ROC) curve and a box plot. As shown in Fig. 5, the area under the curve (AUC) values (> 0.96), which indicate the area under the ROC curve, signify predictive performance with a successful confusion matrix of Fig. 6A (also see Additional file 1: Table S3). Furthermore, the ROC curve shows no significant dependence on accuracy among the ligands classified by the targets. Furthermore, the average precision based on the percentile rank of the KLD features described the distributional information of the predictive model in the box plot (Fig. 7). The patterns in the “RESPONSE” of the RF classifier are shown in the box plot. The horizontal line (orange) shows a skewed decision boundary in the RF classifier, which is inherited from the characteristics of our RAW dataset with an irregular probability density.

**Table 2:** The performance of the RF model in predicting DTI

Target no.	Precision	Recall	F1-score
Q1	0.91	0.93	0.92
Q2	0.97	0.95	0.96
Q3	0.99	0.95	0.97
Q4	0.98	0.95	0.97
Q5	0.88	0.92	0.9
Q6	0.84	0.79	0.81
Q7	0.95	0.97	0.96
Q8	0.83	0.87	0.85
Q9	0.87	0.91	0.89
Q10	0.97	0.9	0.94
Q11	0.76	0.78	0.77
Q12	0.85	0.9	0.87
Q13	1	1	1
Q14	0.81	0.9	0.85
Q15	0.84	0.79	0.81
Q16	0.93	0.92	0.93
Q17	0.79	0.67	0.73

In sequence, we compared the performance of the KLD-RF model with other chemical similarity-based DTI studies (PASS, SEA, CSNAP2D, and CSNAP3D) as shown in Table 3 [5, 24, 25, 47, 56–58]. Despite the difference in the types of used data (target and their ligands), these studies were compared through statistical values, recall, and AUC. Notably, the superiority of KLD-RF over CSNAP3D was observed in the common target HSP90(Q2). Moreover, the performance of network-based methods (CSNAP2D and CSNAP3D) and SEA depends on the similarity cut-off. CSNAP3D cannot consider conformational flexibility. In addition, SEA has the assumption of a probability density function. Now that the utility potential of KLD-RF was presented, we tried to build the KDL-RF model using 2D similarity and out-of-set data (Table 4). While the probability density function of the sigma opioid receptor (Q3) was fitted to a 3D similarity histogram (of 634 conformers), only five ligands were too small to build a 2D histogram of Q3. Thus, the Q–Q matrix of the target was not used and only Q–L vectors between the five ligands and 16 targets were calculated to make 16 KLD feature vectors. Clearly, the average performance of 3D KLD-RF was superior to that of 2D KLD-RF. Moreover, the 3D KLD-RF model was validated by another out-of set, unprecedented bis-N,N-diarylamino tetrahydropyran compounds, which are modulators for Vitamin D receptor (VDR) expression) [26, 50]. In this case, similarly to target Q3 in 2D KLD-RF, the VDR modulators have a target label (Q0) but don’t have a KLD vector for VDR. The out-of-set validation showed comparable performance to the validation of 17 targets.

**Table 3:** Test comparison between the KLD–RF model and DTI prediction models

Model	Test Set	Drug structure (Sim)	Similarity metric	Highest recall	AUC	Refs.
KLD-RF	17 Targets in ChEMBL	Multiple Conformers (3D-Sim)	KLD vector from TC	1.00 Average: 0.889	Average: 0.992 HSP90: 0.998	This Work
CSNAP3D	6 Targets in DUD	One Conformer with Lowest Energy (3D-Sim)	28 including TC with cut-off 0.85	0.98	AUC* 0.54—0.70 HSP90: 0.79	Lo et al. [25]
CSNAP2D**	6 Targets in DUD	2D Structure (2D-Sim)	TC with cut-off 0.6	0.83	–	Lo et al. [24]
SEA**			TC with cut-off 0.57	0.64	0.972***	Keiser et al. [47]
PASS**			Probability Function	0.11	–	Lagunin et al. [56]
SwissTarget	17 Targets in ChEMBL	2D + 3D-Sim	Probability Function from 2 and 3D TC	0.99 Average: 0.748	Average: 0.869	Gfeller et al. [58]

AUC of CSNAP3D*: the average area-under-curve (AUC) was calculated from the curve having rank orders (%) as x-axis and TPR as y-axis. The AUC range was achieved from used different Sim metric. CSNAP2D, SEA, and PASS**: the described performance metric, TPR and AUC were citied from CSNAP3D [25]. The AUC of SEA was citied [59]

**Table 4:** Comparison between KLD–RF model and DTI prediction models

	2D KLD-RF*	3D KLD-RF		
Molecular representation	Morgan 2D	E3FP (Omega Conf)	E3FP (Omega Conf)	E3FP (Rdkit Conf)
Number of KLD feature vectors	16	17	17	17
Number of targets	17	17	17 + 1(out-of-set)	17
Out-of-bag score estimate	0.786	0.876	0.874	0.811
Mean accuracy score	0.794	0.882	0.884	0.815

2D KLD-RF*: Because Sigma opioid receptor of Q3 has 5 ligands not enough to make probability density (2D: 5 ligands vs 3D: 634 conformers), KLD-Q3 feature (KLD feature vector of Q3) was excluded in 2D KLD-RF model and just data of Q3 were included during training/test

### Feature correlation and importance of KLD-based classifier

**Fig. 8:**
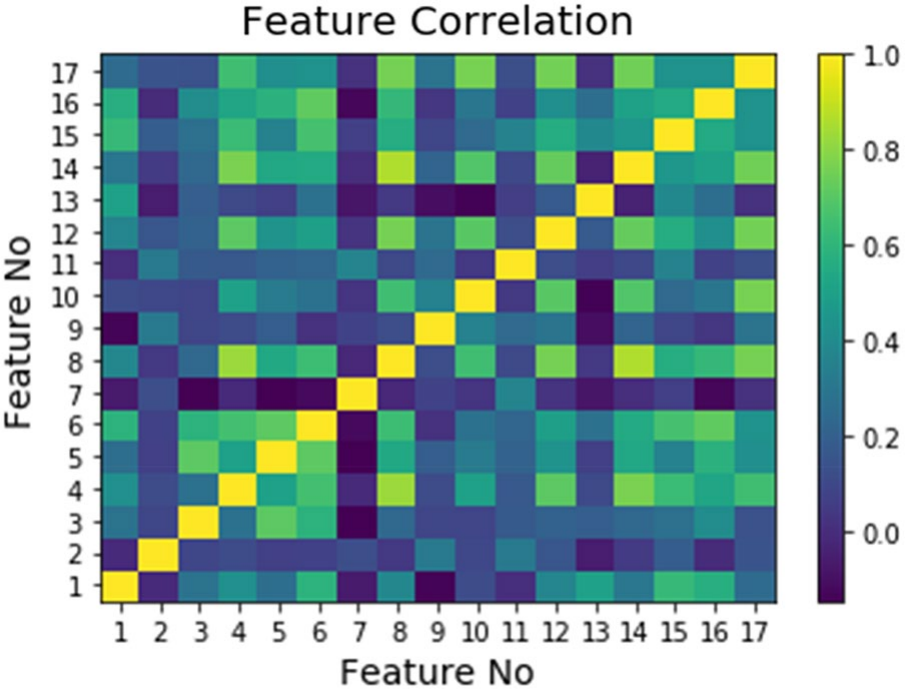
Correlation map between KLD feature vectors in the RF model

**Fig. 9:**
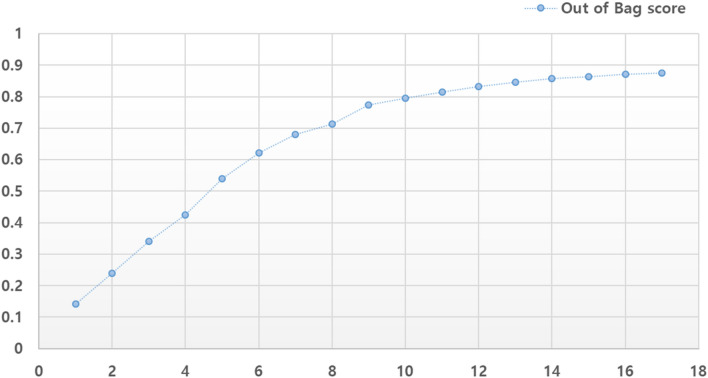
Feature pruning of less important features to show out-of-bag score. X-axis: the number of KLD feature vectors; Y-axis: the accuracy with respect to the number of features

To interpret the DTI model, we conducted a feature analysis of the correlation matrix between features (Fig. 8) and pruned less important features (Fig. 9). In addition to the correlation, the relative importance of a feature in an RF model can be measured with respect to the dependent variable. Figure 8 shows the pairwise correlation coefficients, which reflect the amount of dependence among the features. Each value corresponds to a lower divergence between the q(x) densities of target classes. By providing a criterion for variable selection, a high correlation is achieved among the subset of features, which reduces the importance of such features and hence the prediction accuracy. However, most of the DTI features, except for the 17th feature vector (generated from the ligands of the epidermal growth factor receptor Q17), showed an acceptable correlation coefficient of less than 0.7. Several methods can be used to calculate the feature importance in terms of their effect on the model. The most typical metric, i.e., the mean decrease in impurity, defines the mean impurity reduction as the importance criterion when each feature is deleted in a model. If the corresponding feature value is randomly assigned, then the predicted value become less than the benchmark value, and vice versa. The higher importance of a feature in our study implies the uniqueness of the q(x) density function, which is comparable. Figure 9 illustrates the importance of these features in the DTI model. Generally, pruning less important features is expected to result in higher classification accuracy. In our DTI model, more than 10 features indicated an accuracy exceeding 0.8. Feature selection is vital to model stability and accuracy. Focusing on small numbers of features (10 to 15) is acceptable to avoid dimensionality issues. Because the standard size of the training samples is 15,000 for each target, 10 to 15 features are reasonable to avoid overfitting.

## Conclusion

Herein, we presented an RF model to identify the targets of a drug using KLD vectors. Our novel combination of nonparametric density estimation, KLD, and RF models resulted in an effective chemocentric DTI prediction for drug discovery. Examples showing the use of a new similarity vector and the consideration of the heterogeneity of similarity distributions for reliable DTI predictions were presented. To our best knowledge, this study is the first 3D-chemocentric DTI classifier without a user-defined similarity cut-off. The RF model uses an information metric-designed feature vector to leverage more specific information than our previous approaches. Furthermore, pairwise comparison of ligands and their candidate targets explicitly describes a ligand’s characteristics, which serves as a bridge for an ML classifier. In a computationally limited environment, the dimensions (the size of the feature vectors) can be controlled based on the number of target spaces. In addition to the Jaccard–Tanimoto coefficient, another similarity metric (e.g., the cosine similarity and Soergel similarity) of the descriptor (fingerprint) becomes a proxy for describing a ligand in the context of our methodology. In future studies, we will further clarify this framework based on diverse ML algorithms. In particular, the development of novel unprecedented drugs will be applied to our DTI prediction framework to expand our method to more practical biomedical contexts.

## Supplementary Information

**Additional file 1. ** Supplementary Information File.

## Data Availability

Python code, and refined data will be available in GitHub. https://github.com/college-of-pharmacy-gachon-university/KLD2.
