# Correction : Random-forest model for drug–target interaction prediction via Kullback–Leibler divergence

**DOI:** 10.1186/s13321-022-00653-0

**Published:** 2022-11-02

**Authors:** Sangjin Ahn, Si Eun Lee, Mi-hyun Kim

**Affiliations:** 1 Gachon Institute of Pharmaceutical Science and Department of Pharmacy, College of Pharmacy, Gachon University, 191 Hambakmoeiro, Yeonsu-Gu, Incheon, Republic of Korea; 2 Department of Artificial Intelligence, Ajou University, Suwon, 16499 Republic of Korea

## Correction: Journal of Cheminformatics (2022) 14:67 https://doi.org/10.1186/s13321-022-00644-1

Following publication of the original article [1], the authors identified a spelling in the title and in the body of the text.

Incorrect: Kullbeck.

Correct: Kullback.

The original article has been corrected.
