# Supplementary material for: Random-forest model for drug–target interaction prediction via Kullbeck–Leibler divergence
Source: J Cheminform. 2022 Oct 3;14:67. doi: 10.1186/s13321-022-00644-1 (PMC9531514; doi:10.1186/s13321-022-00644-1)
Supplement: Supplementary file 1 — Additional file 1. Supplementary Information File. [file 13321_2022_644_MOESM1_ESM.docx]

**Supporting Information**

**Random-Forest Model for Drug Target-Interaction Prediction via Kullbeck–Leibler Divergence**

Sangjin Ahn^a,b^, Sieun Lee^a^ and Mi-hyun Kim^a,*^

^a^Gachon Institute of Pharmaceutical Science and Department of Pharmacy, College of Pharmacy, Gachon University, 191 Hambakmoeiro, Yeonsu-gu, Incheon, Republic of Korea

^b^Department of Financial Engineering, College of Business, Ajou University, Suwon, 16499, Republic of Korea

^*^Author for correspondence

E-mail: [kmh0515@gachon.ac.kr](mailto:kmh0515@gachon.ac.kr)

**Table of Contents**

1. Dataset Description(S.Table1)
2. The Overlapped Ligands Among Targets (S.Table2)
3. The Confusion Matrix of KLD-RF Models for Test Data (S.Table3)
4. Computation of Kullbeck-Leibler Divergence Vectors
5. Structural Information of Q-Q Density Models by Each Target (S.Table4, S.Fig1)
6. The Pair-Plot & Robustness for 3D KLD-RF Model (S.Fig2, S.Fig3)
7. Statistical Performance of 2D-RF model in predicting DTI (S.Table5)
8. Statistical Performance of SwissTarget model in predicting DTI (S.Table6)
9. **Dataset Description (S.Table1)**

**Generated conformers using Omega of Openeye**

| Target  Info.  [Q]CHEMBL  (Name) | No. of  Compounds (Raw) | No. of  Compounds*  (Successful Generation of Conformers using Omega) | Conformer Population  Size  (E3FP) | Sampling** |
| --- | --- | --- | --- | --- |
| [Q1] CHEMBL2366517 (HIV-1 Protease) | 660 | **581** | 94421 | **15000** |
| [Q2] CHEMBL4303 (Heat shock protein 90 kDa beta) | 601 | **524** | 84375 | **15000** |
| [Q3] CHEMBL4153 (Sigma opioid receptor) | 8 | **5** | 634 | **634** |
| [Q4] CHEMBL3119 (Transient receptor potential  cation C subfamily V4) | 394 | **363** | 61060 | **15000** |
| [Q5] CHEMBL339 (Dopamine D2 receptor) | 2416 | **2005** | 287494 | **15000** |
| [Q6] CHEMBL274 (CC-Chemokine Receptor 5) | 3813 | **2751** | 429802 | **15000** |
| [Q7] CHEMBL1781 (DNA topoisomerase I) | 511 | **440** | 29493 | **15000** |
| [Q8] CHEMBL5251 (Tyrosine-protein kinase BTK) | 2834 | **2231** | 379529 | **15000** |
| [Q9] CHEMBL1978 (Cytochrome P450 (CYP) 1B1) | 2767 | **2047** | 140781 | **15000** |
| [Q10] CHEMBL4142 (Fibroblast growth factor receptor 1) | 1131 | **1055** | 200052 | **15000** |
| [Q11] CHEMBL2056 (Dopamine D1 receptor) | 1021 | **901** | 67314 | **15000** |
| [Q12] CHEMBL2973 (Rho-associated protein kinase 2) | 2386 | **1836** | 297138 | **15000** |
| [Q13] CHEMBL1667684 (Neuraminidase - Influenza A virus) | 78 | **45** | 8557 | **8557** |
| [Q14] CHEMBL2842 (Serinethreonine-protein kinasemTOR) | 4596 | **4139** | 602995 | **15000** |
| [Q15] CHEMBL204 (Hepsin serine protease) | 3236 | **1777** | 291887 | **15000** |
| [Q16] CHEMBL5023 (p53-binding protein Mdm-2) | 2900 | **2089** | 175930 | **15000** |
| [Q17] CHEMBL203 (Epidermal growth factor receptor) | 12496 | **8936** | 1254040 | **15000** |

**Generated conformers using RDKit^*^**

| Target  Info.  [Q]CHEMBL  (Name) | No. of  Compounds (Raw) | No. of  Compounds  (Successful Generation of Conformers)** | Conformer Population  Size  (E3FP) | Sampling*** |
| --- | --- | --- | --- | --- |
| [Q1] CHEMBL2366517 (HIV-1 Protease) | 660 | **581** | **1551** | **1551** |
| [Q2] CHEMBL4303 (Heat shock protein 90 kDa beta) | 601 | **524** | **1195** | **1195** |
| [Q3] CHEMBL4153 (Sigma opioid receptor) | 8 | **5** | **8** | **8** |
| [Q4] CHEMBL3119 (Transient receptor potential  cation C subfamily V4) | 394 | **363** | **925** | **925** |
| [Q5] CHEMBL339 (Dopamine D2 receptor) | 2416 | **2005** | **3830** | **3830** |
| [Q6] CHEMBL274 (CC-Chemokine Receptor 5) | 3813 | **2751** | **6897** | **6897** |
| [Q7] CHEMBL1781 (DNA topoisomerase I) | 511 | **440** | **764** | **764** |
| [Q8] CHEMBL5251 (Tyrosine-protein kinase BTK) | 2834 | **2231** | **5474** | **5474** |
| [Q9] CHEMBL1978 (Cytochrome P450 (CYP) 1B1) | 2767 | **2047** | **3328** | **3328** |
| [Q10] CHEMBL4142 (Fibroblast growth factor receptor 1) | 1131 | **1055** | **2681** | **2681** |
| [Q11] CHEMBL2056 (Dopamine D1 receptor) | 1021 | **901** | **1554** | **1554** |
| [Q12] CHEMBL2973 (Rho-associated protein kinase 2) | 2386 | **1836** | **4066** | **4066** |
| [Q13] CHEMBL1667684 (Neuraminidase - Influenza A virus) | 78 | **45** | **144** | **144** |
| [Q14] CHEMBL2842 (Serinethreonine-protein kinasemTOR) | 4596 | **4139** | **8857** | **8857** |
| [Q15] CHEMBL204 (Hepsin serine protease) | 3236 | **1777** | **7510** | **7510** |
| [Q16] CHEMBL5023 (p53-binding protein Mdm-2) | 2900 | **2089** | **5191** | **5191** |
| [Q17] CHEMBL203 (Epidermal growth factor receptor) | 12496 | **8936** | **19057** | **19057** |

*The conformers were generated under the condition.

cids = AllChem.EmbedMultipleConfs(molslist[i][j], clearConfs=True, numConfs=3, pruneRmsThresh=1)

**The data was used for 2D similarity calculation.

***The data was used for 3D similarity calculation.

1. **The Overlapped Ligands Among Targets** **(S.Table2)**

|  | **Q1** | **Q2** | **Q3** | **Q4** | **Q5** | **Q6** | **Q7** | **Q8** | **Q9** | **Q10** | **Q11** | **Q12** | **Q13** | **Q14** | **Q15** | **Q16** | **Q17** |
| --- | --- | --- | --- | --- | --- | --- | --- | --- | --- | --- | --- | --- | --- | --- | --- | --- | --- |
| **Q1** | Self | 0 | 0 | 0 | 0 | 5 | 0 | 0 | 3 | 0 | 5 | 0 | 0 | 0 | 0 | 0 | 6 |
| **Q2** | 0 | Self | 0 | 0 | 0 | 0 | 1 | 0 | 1 | 1 | 0 | 1 | 0 | 1 | 0 | 0 | 1 |
| **Q3** | 0 | 0 | Self | 0 | 2 | 1 | 0 | 0 | 0 | 0 | 1 | 0 | 0 | 0 | 0 | 0 | 1 |
| **Q4** | 0 | 0 | 0 | Self | 0 | 0 | 0 | 0 | 0 | 0 | 0 | 0 | 0 | 0 | 0 | 0 | 0 |
| **Q5** | 0 | 0 | 2 | 0 | Self | 24 | 1 | 0 | 0 | 0 | 37 | 0 | 0 | 0 | 0 | 5 | 24 |
| **Q6** | 11 | 0 | 2 | 0 | 104 | Self | 33 | 1 | 112 | 0 | 874 | 6 | 0 | 20 | 1 | 2 | 898 |
| **Q7** | 0 | 1 | 0 | 0 | 1 | 7 | Self | 0 | 2 | 0 | 7 | 0 | 0 | 0 | 0 | 0 | 11 |
| **Q8** | 0 | 0 | 0 | 0 | 0 | 1 | 0 | Self | 0 | 24 | 1 | 25 | 0 | 25 | 0 | 0 | 233 |
| **Q9** | 2 | 1 | 0 | 0 | 0 | 14 | 2 | 0 | Self | 0 | 14 | 0 | 0 | 0 | 2 | 0 | 24 |
| **Q10** | 0 | 1 | 0 | 0 | 0 | 0 | 0 | 64 | 0 | Self | 0 | 14 | 0 | 4 | 0 | 0 | 113 |
| **Q11** | 11 | 0 | 2 | 0 | 113 | 871 | 33 | 1 | 112 | 0 | Self | 6 | 0 | 20 | 1 | 2 | 899 |
| **Q12** | 0 | 1 | 0 | 0 | 0 | 1 | 0 | 29 | 0 | 18 | 1 | Self | 0 | 5 | 0 | 0 | 141 |
| **Q13** | 0 | 0 | 0 | 0 | 0 | 0 | 0 | 0 | 0 | 0 | 0 | 0 | Self | 0 | 0 | 0 | 0 |
| **Q14** | 0 | 1 | 0 | 0 | 0 | 5 | 0 | 29 | 0 | 9 | 5 | 6 | 0 | Self | 0 | 0 | 407 |
| **Q15** | 0 | 0 | 0 | 0 | 0 | 1 | 0 | 0 | 2 | 0 | 1 | 0 | 0 | 0 | Self | 0 | 4 |
| **Q16** | 0 | 0 | 0 | 0 | 20 | 2 | 0 | 0 | 0 | 0 | 2 | 0 | 0 | 0 | 0 | Self | 2 |
| **Q17** | 13 | 1 | 2 | 0 | 104 | 871 | 34 | 233 | 127 | 81 | 875 | 83 | 0 | 283 | 3 | 0 | Self |

1. **The Confusion Matrix for Test Data (S.Table3)**

**3D KLD-RF Model**

|  | **Q1** | **Q2** | **Q3** | **Q4** | **Q5** | **Q6** | **Q7** | **Q8** | **Q9** | **Q10** | **Q11** | **Q12** | **Q13** | **Q14** | **Q15** | **Q16** | **Q17** |
| --- | --- | --- | --- | --- | --- | --- | --- | --- | --- | --- | --- | --- | --- | --- | --- | --- | --- |
| **Q1** | 2759 | 0 | 0 | 0 | 3 | 27 | 9 | 2 | 19 | 0 | 21 | 2 | 0 | 6 | 91 | 12 | 3 |
| **Q2** | 1 | 2948 | 0 | 1 | 26 | 4 | 5 | 11 | 17 | 2 | 2 | 33 | 0 | 15 | 10 | 3 | 23 |
| **Q3** | 0 | 0 | 133 | 0 | 7 | 0 | 0 | 0 | 0 | 0 | 1 | 0 | 0 | 0 | 0 | 0 | 0 |
| **Q4** | 0 | 2 | 0 | 2913 | 12 | 14 | 1 | 11 | 15 | 0 | 7 | 9 | 0 | 54 | 14 | 3 | 3 |
| **Q5** | 2 | 5 | 2 | 8 | 2744 | 40 | 4 | 9 | 17 | 0 | 52 | 26 | 0 | 10 | 17 | 19 | 13 |
| **Q6** | 41 | 3 | 0 | 4 | 53 | 2361 | 9 | 17 | 20 | 0 | 309 | 20 | 0 | 37 | 48 | 43 | 14 |
| **Q7** | 20 | 3 | 0 | 0 | 3 | 0 | 2895 | 1 | 22 | 0 | 26 | 1 | 0 | 2 | 5 | 2 | 10 |
| **Q8** | 1 | 5 | 0 | 6 | 7 | 24 | 4 | 2556 | 11 | 28 | 1 | 44 | 0 | 92 | 18 | 8 | 135 |
| **Q9** | 27 | 4 | 0 | 3 | 24 | 16 | 44 | 2 | 2778 | 0 | 59 | 35 | 0 | 25 | 20 | 2 | 12 |
| **Q10** | 0 | 1 | 0 | 0 | 4 | 15 | 2 | 79 | 4 | 2688 | 0 | 52 | 0 | 28 | 10 | 6 | 101 |
| **Q11** | 65 | 13 | 0 | 3 | 141 | 128 | 35 | 6 | 114 | 0 | 2349 | 11 | 0 | 17 | 58 | 19 | 52 |
| **Q12** | 1 | 8 | 0 | 3 | 17 | 26 | 8 | 37 | 23 | 5 | 4 | 2731 | 0 | 69 | 32 | 9 | 48 |
| **Q13** | 0 | 0 | 0 | 0 | 0 | 0 | 0 | 0 | 0 | 0 | 0 | 0 | 1759 | 0 | 0 | 0 | 0 |
| **Q14** | 2 | 7 | 0 | 5 | 7 | 23 | 3 | 58 | 16 | 3 | 6 | 50 | 0 | 2616 | 33 | 10 | 75 |
| **Q15** | 84 | 10 | 0 | 4 | 47 | 66 | 13 | 20 | 71 | 0 | 58 | 68 | 0 | 108 | 2359 | 45 | 30 |
| **Q16** | 27 | 4 | 0 | 0 | 2 | 42 | 5 | 37 | 5 | 0 | 9 | 18 | 0 | 24 | 40 | 2790 | 16 |
| **Q17** | 12 | 14 | 0 | 10 | 26 | 30 | 24 | 239 | 74 | 32 | 182 | 122 | 0 | 143 | 69 | 30 | 2054 |

**2D KLD-RF Model**

|  | **Q1** | **Q2** | **Q3** | **Q4** | **Q5** | **Q6** | **Q7** | **Q8** | **Q9** | **Q10** | **Q11** | **Q12** | **Q13** | **Q14** | **Q15** | **Q16** | **Q17** |
| --- | --- | --- | --- | --- | --- | --- | --- | --- | --- | --- | --- | --- | --- | --- | --- | --- | --- |
| **Q1** | 125 | 0 | 0 | 0 | 0 | 11 | 1 | 0 | 6 | 0 | 0 | 0 | 0 | 0 | 0 | 0 | 13 |
| **Q2** | 0 | 70 | 0 | 0 | 8 | 4 | 0 | 2 | 8 | 0 | 1 | 3 | 0 | 5 | 0 | 1 | 34 |
| **Q3** | 0 | 0 | 0 | 0 | 0 | 1 | 0 | 0 | 0 | 0 | 0 | 0 | 0 | 0 | 0 | 0 | 0 |
| **Q4** | 0 | 0 | 0 | 70 | 0 | 1 | 0 | 3 | 0 | 0 | 0 | 1 | 0 | 0 | 0 | 0 | 3 |
| **Q5** | 0 | 0 | 0 | 0 | 439 | 22 | 0 | 0 | 7 | 0 | 4 | 3 | 0 | 5 | 0 | 4 | 34 |
| **Q6** | 2 | 1 | 1 | 0 | 18 | 485 | 1 | 1 | 7 | 0 | 80 | 2 | 0 | 2 | 1 | 0 | 115 |
| **Q7** | 1 | 0 | 0 | 0 | 2 | 1 | 66 | 0 | 9 | 0 | 0 | 0 | 0 | 1 | 0 | 0 | 31 |
| **Q8** | 0 | 0 | 0 | 0 | 0 | 0 | 0 | 434 | 1 | 4 | 0 | 7 | 0 | 24 | 0 | 0 | 98 |
| **Q9** | 3 | 2 | 0 | 0 | 8 | 5 | 2 | 0 | 402 | 0 | 0 | 2 | 0 | 8 | 0 | 4 | 66 |
| **Q10** | 0 | 0 | 0 | 0 | 0 | 2 | 0 | 3 | 3 | 195 | 0 | 2 | 0 | 5 | 0 | 0 | 47 |
| **Q11** | 1 | 1 | 0 | 0 | 10 | 92 | 1 | 0 | 7 | 0 | 12 | 1 | 0 | 2 | 1 | 2 | 104 |
| **Q12** | 0 | 0 | 0 | 0 | 2 | 6 | 0 | 6 | 3 | 0 | 0 | 368 | 0 | 19 | 0 | 0 | 53 |
| **Q13** | 0 | 0 | 0 | 0 | 0 | 0 | 0 | 0 | 0 | 0 | 0 | 0 | 10 | 0 | 5 | 0 | 0 |
| **Q14** | 0 | 0 | 0 | 0 | 0 | 11 | 0 | 11 | 7 | 0 | 0 | 3 | 0 | 885 | 0 | 1 | 108 |
| **Q15** | 0 | 0 | 0 | 0 | 0 | 0 | 0 | 0 | 0 | 0 | 0 | 0 | 0 | 0 | 744 | 0 | 2 |
| **Q16** | 6 | 0 | 0 | 0 | 7 | 7 | 0 | 0 | 5 | 0 | 0 | 1 | 0 | 2 | 0 | 495 | 37 |
| **Q17** | 1 | 2 | 1 | 0 | 12 | 108 | 0 | 46 | 28 | 12 | 89 | 36 | 0 | 97 | 1 | 5 | 1842 |

**3D KLD-RF Model with Out-of-Set VDR**

|  | **VDR** | **Q1** | **Q2** | **Q3** | **Q4** | **Q5** | **Q6** | **Q7** | **Q8** | **Q9** | **Q10** | **Q11** | **Q12** | **Q13** | **Q14** | **Q15** | **Q16** | **Q17** |
| --- | --- | --- | --- | --- | --- | --- | --- | --- | --- | --- | --- | --- | --- | --- | --- | --- | --- | --- |
| **VDR** | 24 | 0 | 0 | 0 | 0 | 0 | 0 | 0 | 0 | 0 | 0 | 0 | 0 | 0 | 0 | 0 | 0 | 0 |
| **Q1** | 0 | 2767 | 3 | 0 | 0 | 1 | 27 | 8 | 1 | 24 | 0 | 27 | 1 | 0 | 5 | 79 | 15 | 0 |
| **Q2** | 0 | 3 | 2863 | 0 | 1 | 26 | 4 | 3 | 7 | 16 | 1 | 3 | 30 | 0 | 10 | 9 | 3 | 24 |
| **Q3** | 0 | 0 | 0 | 133 | 0 | 5 | 0 | 0 | 0 | 0 | 0 | 0 | 0 | 0 | 0 | 0 | 0 | 0 |
| **Q4** | 0 | 0 | 0 | 0 | 2908 | 14 | 20 | 1 | 11 | 19 | 0 | 6 | 10 | 0 | 43 | 11 | 3 | 6 |
| **Q5** | 0 | 3 | 9 | 3 | 6 | 2747 | 48 | 2 | 10 | 16 | 0 | 60 | 25 | 0 | 6 | 18 | 15 | 11 |
| **Q6** | 0 | 35 | 4 | 1 | 2 | 51 | 2369 | 8 | 12 | 20 | 1 | 324 | 21 | 0 | 33 | 50 | 28 | 13 |
| **Q7** | 0 | 21 | 0 | 0 | 0 | 1 | 2 | 2918 | 0 | 27 | 1 | 34 | 2 | 0 | 1 | 12 | 3 | 7 |
| **Q8** | 0 | 1 | 2 | 0 | 4 | 5 | 15 | 1 | 2566 | 7 | 22 | 3 | 47 | 0 | 100 | 23 | 9 | 102 |
| **Q9** | 0 | 23 | 3 | 0 | 2 | 23 | 14 | 35 | 2 | 2798 | 1 | 62 | 27 | 0 | 22 | 28 | 4 | 16 |
| **Q10** | 0 | 0 | 2 | 0 | 0 | 3 | 11 | 1 | 84 | 9 | 2759 | 2 | 44 | 0 | 35 | 4 | 4 | 104 |
| **Q11** | 0 | 73 | 13 | 0 | 0 | 157 | 120 | 31 | 5 | 99 | 0 | 2291 | 3 | 0 | 14 | 47 | 17 | 59 |
| **Q12** | 0 | 1 | 12 | 0 | 4 | 13 | 23 | 2 | 46 | 15 | 7 | 4 | 2659 | 0 | 60 | 19 | 10 | 46 |
| **Q13** | 0 | 0 | 0 | 0 | 0 | 0 | 0 | 0 | 0 | 0 | 0 | 0 | 0 | 1789 | 0 | 0 | 0 | 0 |
| **Q14** | 0 | 3 | 4 | 0 | 4 | 12 | 37 | 2 | 57 | 12 | 2 | 8 | 38 | 0 | 2660 | 31 | 10 | 93 |
| **Q15** | 0 | 91 | 13 | 0 | 3 | 32 | 92 | 14 | 28 | 53 | 1 | 56 | 87 | 0 | 106 | 2401 | 41 | 34 |
| **Q16** | 0 | 29 | 6 | 0 | 0 | 5 | 40 | 5 | 44 | 6 | 0 | 11 | 25 | 0 | 21 | 52 | 2679 | 24 |
| **Q17** | 0 | 11 | 17 | 0 | 7 | 23 | 47 | 29 | 219 | 81 | 32 | 162 | 111 | 0 | 140 | 60 | 18 | 2107 |

**3D KLD-RF Model (using RDKit Generated Conformers)**

|  | **Q1** | **Q2** | **Q3** | **Q4** | **Q5** | **Q6** | **Q7** | **Q8** | **Q9** | **Q10** | **Q11** | **Q12** | **Q13** | **Q14** | **Q15** | **Q16** | **Q17** |
| --- | --- | --- | --- | --- | --- | --- | --- | --- | --- | --- | --- | --- | --- | --- | --- | --- | --- |
| **Q1** | 242 | 0 | 0 | 0 | 3 | 14 | 6 | 0 | 13 | 0 | 0 | 0 | 0 | 0 | 85 | 0 | 16 |
| **Q2** | 0 | 214 | 0 | 1 | 12 | 7 | 0 | 5 | 10 | 0 | 2 | 11 | 0 | 8 | 12 | 2 | 30 |
| **Q3** | 0 | 0 | 0 | 0 | 6 | 0 | 0 | 0 | 0 | 0 | 0 | 0 | 0 | 0 | 0 | 0 | 0 |
| **Q4** | 0 | 0 | 0 | 165 | 6 | 11 | 0 | 3 | 3 | 1 | 0 | 5 | 0 | 1 | 2 | 3 | 15 |
| **Q5** | 0 | 1 | 0 | 0 | 755 | 61 | 0 | 6 | 15 | 6 | 3 | 22 | 0 | 8 | 24 | 11 | 61 |
| **Q6** | 11 | 0 | 0 | 1 | 48 | 1193 | 1 | 19 | 46 | 0 | 69 | 15 | 0 | 21 | 115 | 17 | 148 |
| **Q7** | 7 | 0 | 0 | 0 | 1 | 7 | 97 | 1 | 7 | 0 | 1 | 0 | 0 | 3 | 15 | 6 | 41 |
| **Q8** | 1 | 2 | 0 | 0 | 0 | 8 | 0 | 1073 | 7 | 7 | 2 | 32 | 0 | 50 | 15 | 5 | 132 |
| **Q9** | 6 | 1 | 0 | 0 | 20 | 19 | 5 | 3 | 640 | 1 | 5 | 13 | 0 | 10 | 29 | 7 | 80 |
| **Q10** | 0 | 0 | 0 | 0 | 0 | 2 | 0 | 17 | 3 | 542 | 0 | 13 | 0 | 10 | 7 | 0 | 72 |
| **Q11** | 8 | 3 | 0 | 0 | 36 | 132 | 1 | 0 | 34 | 1 | 40 | 5 | 0 | 5 | 35 | 5 | 79 |
| **Q12** | 0 | 0 | 0 | 1 | 4 | 19 | 0 | 12 | 17 | 1 | 1 | 810 | 0 | 32 | 23 | 0 | 92 |
| **Q13** | 2 | 0 | 0 | 0 | 0 | 0 | 0 | 0 | 0 | 0 | 0 | 0 | 35 | 0 | 4 | 0 | 0 |
| **Q14** | 1 | 0 | 0 | 0 | 3 | 11 | 0 | 24 | 6 | 1 | 2 | 11 | 0 | 2130 | 6 | 10 | 92 |
| **Q15** | 15 | 1 | 0 | 0 | 22 | 82 | 3 | 13 | 34 | 2 | 4 | 50 | 0 | 34 | 1478 | 14 | 139 |
| **Q16** | 5 | 1 | 0 | 0 | 3 | 38 | 0 | 21 | 21 | 0 | 1 | 6 | 0 | 8 | 38 | 1048 | 55 |
| **Q17** | 6 | 1 | 0 | 0 | 14 | 73 | 2 | 57 | 17 | 16 | 15 | 28 | 0 | 44 | 73 | 15 | 4411 |

**SwissTarget Model***

|  | **Q1** | **Q2** | **Q3** | **Q4** | **Q5** | **Q6** | **Q7** | **Q8** | **Q9** | **Q10** | **Q11** | **Q12** | **Q13** | **Q14** | **Q15** | **Q16** | **Q17** |
| --- | --- | --- | --- | --- | --- | --- | --- | --- | --- | --- | --- | --- | --- | --- | --- | --- | --- |
| **Q1** | 229 | 0 | 0 | 0 | 3 | 27 | 0 | 0 | 27 | 0 | 4 | 1 | 0 | 1 | 18 | 2 | 21 |
| **Q2** | 0 | 436 | 0 | 0 | 13 | 3 | 1 | 1 | 12 | 1 | 1 | 11 | 0 | 9 | 9 | 0 | 26 |
| **Q3** | 0 | 0 | 0 | 0 | 3 | 0 | 0 | 0 | 0 | 1 | 1 | 0 | 0 | 0 | 0 | 0 | 0 |
| **Q4** | 0 | 1 | 0 | 307 | 1 | 5 | 0 | 1 | 9 | 0 | 3 | 5 | 0 | 6 | 0 | 1 | 15 |
| **Q5** | 0 | 0 | 0 | 0 | 1701 | 107 | 0 | 7 | 3 | 0 | 2 | 1 | 0 | 2 | 2 | 14 | 145 |
| **Q6** | 0 | 0 | 0 | 0 | 6 | 1971 | 0 | 1 | 7 | 0 | 3 | 0 | 0 | 12 | 15 | 1 | 669 |
| **Q7** | 4 | 0 | 0 | 0 | 5 | 31 | 252 | 2 | 14 | 0 | 5 | 6 | 0 | 0 | 7 | 3 | 45 |
| **Q8** | 0 | 0 | 0 | 1 | 1 | 5 | 0 | 1605 | 0 | 2 | 0 | 11 | 0 | 20 | 10 | 0 | 364 |
| **Q9** | 0 | 0 | 0 | 0 | 7 | 113 | 0 | 0 | 1665 | 0 | 0 | 8 | 0 | 10 | 29 | 0 | 182 |
| **Q10** | 0 | 0 | 0 | 0 | 1 | 3 | 0 | 15 | 1 | 901 | 0 | 7 | 0 | 10 | 2 | 1 | 102 |
| **Q11** | 3 | 0 | 0 | 0 | 73 | 287 | 0 | 3 | 48 | 0 | 97 | 8 | 0 | 8 | 32 | 7 | 286 |
| **Q12** | 0 | 0 | 0 | 0 | 4 | 19 | 0 | 11 | 0 | 1 | 2 | 1609 | 0 | 21 | 11 | 1 | 145 |
| **Q13** | 0 | 0 | 0 | 0 | 0 | 1 | 0 | 0 | 0 | 0 | 2 | 0 | 58 | 0 | 1 | 0 | 2 |
| **Q14** | 0 | 0 | 0 | 0 | 0 | 3 | 0 | 13 | 3 | 1 | 0 | 2 | 0 | 3671 | 3 | 1 | 350 |
| **Q15** | 0 | 0 | 0 | 0 | 4 | 42 | 0 | 2 | 13 | 0 | 1 | 7 | 0 | 36 | 2167 | 7 | 374 |
| **Q16** | 0 | 0 | 0 | 0 | 0 | 24 | 0 | 0 | 0 | 0 | 0 | 0 | 0 | 9 | 10 | 1793 | 51 |
| **Q17** | 1 | 0 | 0 | 0 | 2 | 28 | 0 | 5 | 3 | 0 | 0 | 1 | 0 | 12 | 7 | 1 | 8444 |

* *Scoring Function* **(***s_1_, s_2_***)***=***(***1+***exp[***-a_0_-a_1_s_1_-a_2_s_2_***])***^−1^*

s1 = 2D similarity, s2 = 3D similarity

*a_0_, a_1_, a_2_* were decided from supplementary information of the literature of *Nucleic Acids Research* 42, W32–W38 (2014).

Predicted class of each query was assigned based on the highest ranked probability value calculated from scoring function. In detail, each query can have 31,164 query-ligand pairs and give 31,164 probability values from the scoring function. Thus, the ligand pair showing the highest possibility value was chosen and the target of the ligand was assigned to predicted class of the query.

1. **Computation of Kullbeck-Leibler Divergence Vectors**

Let p(x) be a density from Q-L vector and q(x) be from Q-Q matrix,

The Kullbeck-Leibler Divergence(cite) for nonparametric density is defined by:

$$D\left( P\left\| Q \right. \right) := \int_{-\infty}^{\infty} \ln\left( \frac{p\left( x \right)}{q\left( x \right)} \right)p\left( x \right)dx$$

for x_i in (0, 1) where x1, …x_100

For each p(x), we obtain 17 divergence calculated by candidate targets, The divergence vector for each ligand is labeled by their own target as shown in the next examples.

| **Drugs** | **KLDQ1** | **KLDQ2** | **KLDQ3** | **KLDQ4** | **KLDQ5** | **KLDQ6** | **KLDQ7** | **KLDQ8** | **KLDQ9** | **KLDQ10** | **KLDQ11** | **KLDQ12** | **KLDQ13** | **KLDQ14** | **KLDQ15** | **KLDQ16** | **KLDQ17** |
| --- | --- | --- | --- | --- | --- | --- | --- | --- | --- | --- | --- | --- | --- | --- | --- | --- | --- |
| CHEMBL3126454_0 | 0.59333 | 0.903644 | 3.847702 | 2.2452 | 1.353567 | 0.88767 | 0.124954 | 2.457438 | 0.622791 | 3.606968 | 0.161093 | 2.3451 | 5.951562 | 2.443589 | 0.83235 | 1.076318 | 1.389519 |
| CHEMBL3126453_0 | 0.319358 | 0.617718 | 2.163279 | 1.448611 | 1.319439 | 0.668798 | 0.100405 | 1.80478 | 0.476255 | 3.324672 | 0.194852 | 2.110077 | 7.222029 | 1.983163 | 0.519279 | 0.566461 | 1.050486 |
| CHEMBL3799995_0 | 0.617898 | 0.779734 | 4.100718 | 2.2965 | 1.803264 | 1.214501 | 0.230805 | 3.475001 | 0.780587 | 3.876629 | 0.072032 | 1.278461 | 6.73808 | 2.889051 | 0.628621 | 1.773783 | 1.152712 |
| CHEMBL572988_0 | 0.36622 | 0.369239 | 2.353694 | 1.253695 | 1.11142 | 0.769719 | 0.531816 | 1.881659 | 0.1645 | 2.137363 | 0.348618 | 1.439828 | 7.307991 | 1.854418 | 0.576496 | 1.317806 | 1.165088 |
| CHEMBL556509_0 | 0.223933 | 0.196037 | 1.443353 | 0.320509 | 0.428653 | 0.105264 | 0.232352 | 0.264721 | 0.163272 | 1.069166 | 0.09811 | 0.157102 | 5.023408 | 0.239203 | 0.119151 | 0.353498 | 0.319624 |

1. **Structural Information of Q-Q density models by each target (S.Table4, S.Fig1)**

**S.Table4) The Table of Descriptive statistics by Targets**

| **Target** | **KDE Model (Mean-Std)** |
| --- | --- |
| [Q1] CHEMBL2366517 (HIV-1 Protease) | 0.127-0.051 |
| [Q2] CHEMBL4303 (Heat shock protein 90 kDa beta) | 0.126-0.070 |
| [Q3] CHEMBL4153 (Sigma opioid receptor) | 0.195-0.128 |
| [Q4] CHEMBL3119 (Transient receptor potential cation C subfamily V4) | 0.154-0.076 |
| [Q5] CHEMBL339 (Dopamine D2 receptor) | 0.124-0.045 |
| [Q6] CHEMBL274 (CC-Chemokine Receptor 5) | 0.123-0.041 |
| [Q7] CHEMBL1781 (DNA topoisomerase I) | 0.113-0.078 |
| [Q8] CHEMBL5251 (Tyrosine-protein kinase BTK) | 0.139-0.045 |
| [Q9] CHEMBL1978 (Cytochrome P450 (CYP) 1B1) | 0.108-0.049 |
| [Q10] CHEMBL4142 (Fibroblast growth factor receptor 1) | 0.185-0.086 |
| [Q11] CHEMBL2056 (Dopamine D1 receptor) | 0.098-0.040 |
| [Q12] CHEMBL2973 (Rho-associated protein kinase 2) | 0.145-0.054 |
| [Q13] CHEMBL1667684 (Neuraminidase - Influenza A virus) | 0.3-0.077 |
| [Q14] CHEMBL2842 (Serinethreonine-protein kinasemTOR) | 0.140-0.052 |
| [Q15] CHEMBL204 (Hepsin serine protease) | 0.117-0.039 |
| [Q16] CHEMBL5023 (p53-binding protein Mdm-2) | 0.132-0.053 |
| [Q17] CHEMBL203 (Epidermal growth factor receptor) | 0.126-0.044 |

**S.Fig1) The Overall Density Structure re of 17 Q-Q Matrix**


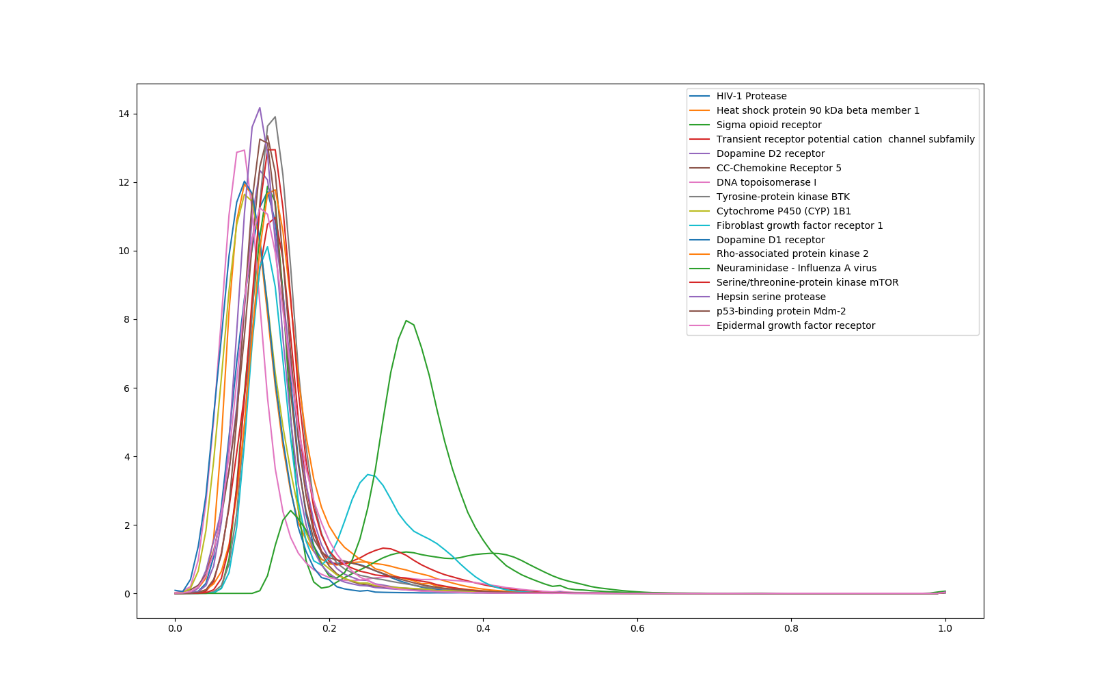


1. **The Pair-plot & Robustness for 3D KLD-RF Model (S.Fig2, S.Fig3)**

**S.Fig2) The pair-plots, which can be obtained from the Random Forest model, represent the detailed information of classifier.**


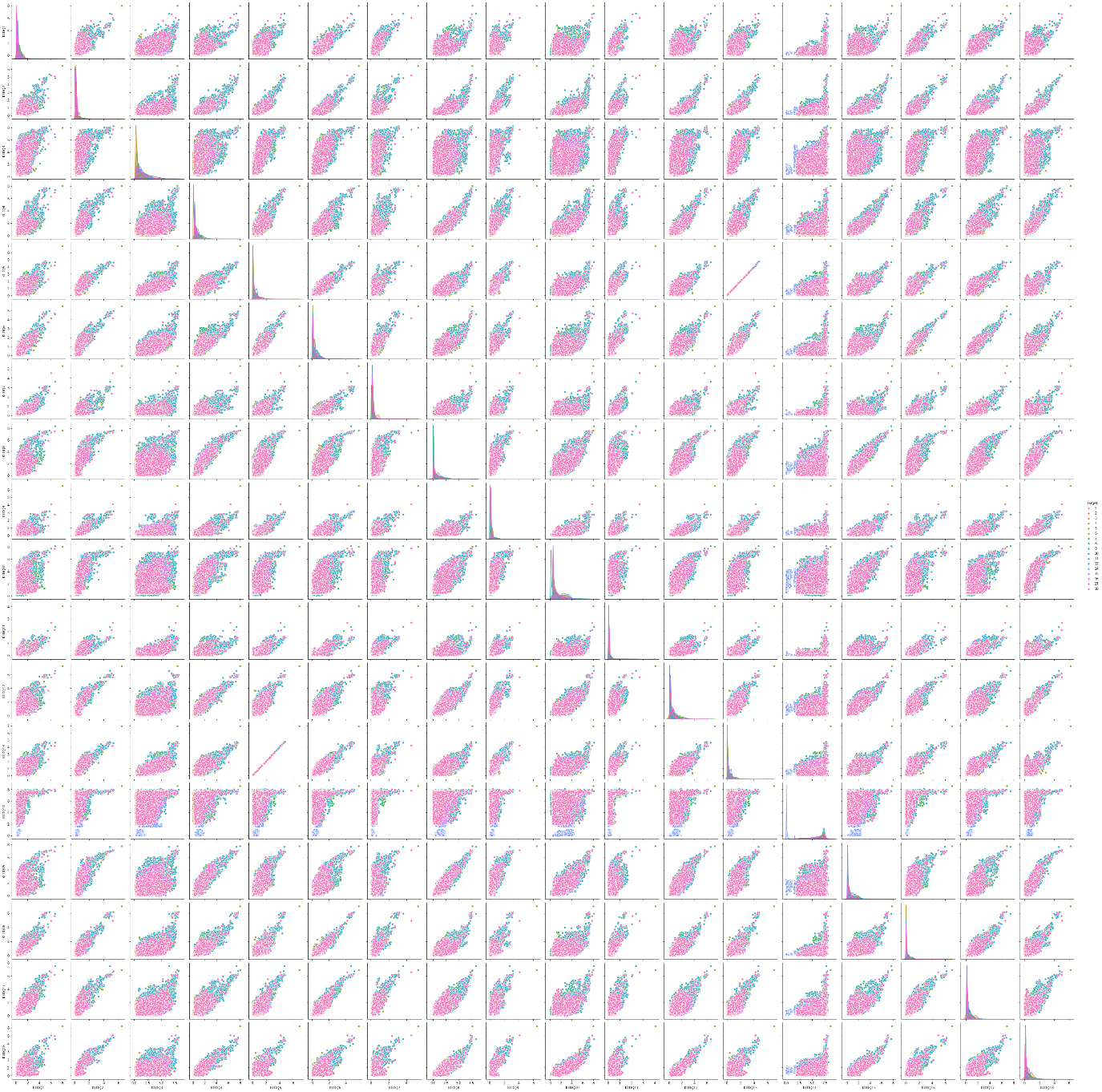


**S.Fig3) Robustness of KLD-RF Model with Radom 10 Seeds.**


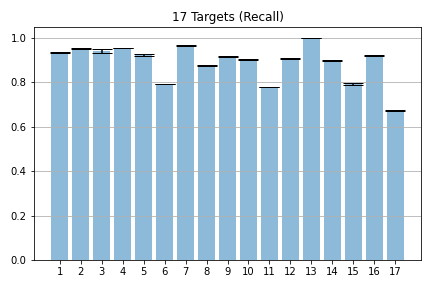


1. **Statistical Performance of 2D-RF model in predicting DTI (S.Table5)**

| Target No. | Precision | Recall | F1-score |
| --- | --- | --- | --- |
| Q1 | 0.9 | 0.8 | 0.85 |
| Q2 | 0.92 | 0.51 | 0.66 |
| Q3* | - | - | - |
| Q4 | 1 | 0.9 | 0.95 |
| Q5 | 0.87 | 0.85 | 0.86 |
| Q6 | 0.64 | 0.68 | 0.66 |
| Q7 | 0.93 | 0.59 | 0.73 |
| Q8 | 0.86 | 0.76 | 0.81 |
| Q9 | 0.82 | 0.8 | 0.81 |
| Q10 | 0.92 | 0.76 | 0.83 |
| Q11 | 0.06 | 0.05 | 0.06 |
| Q12 | 0.86 | 0.81 | 0.83 |
| Q13 | 1 | 0.67 | 0.8 |
| Q14 | 0.84 | 0.86 | 0.85 |
| Q15 | 0.99 | 1 | 0.99 |
| Q16 | 0.97 | 0.88 | 0.92 |
| Q17 | 0.71 | 0.81 | 0.76 |

Test set of Q* is one data.

1. **Statistical Performance of SwissTarget model in predicting DTI (S.Table6)**

| Target No. | Precision | | Recall | | F1-score | |
| --- | --- | --- | --- | --- | --- | --- |
|  | SwissTarget | 3D-KLD | SwissTarget | 3D-KLD | SwissTarget | 3D-KLD |
| Q1 | 0.97 | 0.91 | **0.69** | 0.93 | **0.80** | 0.92 |
| Q2 | 1.00 | 0.97 | **0.83** | 0.95 | **0.91** | 0.96 |
| Q3 | **NA*** | 0.99 | **0.00** | 0.95 | **NA*** | 0.97 |
| Q4 | 1.00 | 0.98 | **0.87** | 0.95 | **0.93** | 0.97 |
| Q5 | 0.93 | 0.88 | **0.86** | 0.92 | **0.89** | 0.9 |
| Q6 | 0.74 | 0.84 | **0.73** | 0.79 | **0.74** | 0.81 |
| Q7 | 1.00 | 0.95 | **0.67** | 0.97 | **0.80** | 0.96 |
| Q8 | 0.96 | 0.83 | **0.79** | 0.87 | **0.87** | 0.85 |
| Q9 | 0.92 | 0.87 | **0.83** | 0.91 | **0.87** | 0.89 |
| Q10 | 0.99 | 0.97 | **0.86** | 0.9 | **0.92** | 0.94 |
| Q11 | 0.80 | 0.76 | **0.11** | 0.78 | **0.20** | 0.77 |
| Q12 | 0.96 | 0.85 | **0.88** | 0.9 | **0.92** | 0.87 |
| Q13 | 1.00 | 1 | **0.91** | 1 | **0.95** | 1 |
| Q14 | 0.96 | 0.81 | **0.91** | 0.9 | **0.93** | 0.85 |
| Q15 | 0.93 | 0.84 | **0.82** | 0.79 | **0.87** | 0.81 |
| Q16 | 0.98 | 0.93 | **0.95** | 0.92 | **0.96** | 0.93 |
| Q17 | 0.75 | 0.79 | **0.99** | 0.67 | **0.86** | 0.73 |

Q3 has TP=FP=0 and FN=5, TN=31,160. Thus, Precision and F1 score are not available.
